# Four New Insecticidal Xanthene Derivatives from the Mangrove-Derived Fungus *Penicillium* sp. JY246

**DOI:** 10.3390/md17120649

**Published:** 2019-11-20

**Authors:** Meng Bai, Cai-Juan Zheng, Xu-Hua Nong, Xue-Ming Zhou, You-Ping Luo, Guang-Ying Chen

**Affiliations:** 1 Key Laboratory of Tropical Medicinal Resource Chemistry of Ministry of Education, Hainan Normal University, Haikou, Hainan 571127, China; xxbai2014@163.com (M.B.); caijuan2002@163.com (C.-J.Z.); nongxuhua4883@163.com (X.-H.N.); xueming2009211@126.com (X.-M.Z.); dengpengfei@gmail.com (Y.-P.L.); 2 Key Laboratory of Tropical Medicinal Plant Chemistry of Hainan Province, College of Chemistry and Chemical Engineering, Hainan Normal University, Haikou, Hainan 571127, China

**Keywords:** *Penicillium* sp., *Ceriops tagal*, xanthene, insecticidal activity

## Abstract

Four new xanthene derivatives, penicixanthenes A–D (**1**–**4**), and one known compound **5** were isolated from a marine mangrove endophytic fungus *Penicillium* sp. JY246 that was obtained from the stem of *Ceriops tagal*. Their structures were determined by detailed NMR, MS spectroscopic data, modified Mosher’s method, and calculated electronic circular dichroism data. All of the isolated compounds were examined for insecticidal activity. Compounds **2** and **3** showed growth inhibition activity against newly hatched larvae of *Helicoverpa armigera* Hubner with the IC_50_ values 100 and 200 μg/mL, respectively, and compounds **1**, **3**, and **4** showed insecticidal activity against newly hatched larvae of *Culex quinquefasciatus* with LC_50_ values of 38.5 (±1.16), 11.6 (±0.58), and 20.5 (±1) μg/mL, respectively. The four xanthene derivatives have the potential to be developed as new biopesticides.

## 1. Introduction

Fungal secondary metabolites have always been considered an important source for drug discovery due to their diverse chemical structures and bioactivities [1]. Among them, *Penicillium* are recognized as important producers of structurally unusual natural products, especially of terpenes with pharmaceutical potential as illustrated with chrysogenester, an anti-inflammatory meroterpenoid-type derivative [2], shearilicine, a cytotoxic indole-diterpenoid possessing a rare carbazole unit [3,4], and the penerpenes, unusual indole-terpenoids which have shown potent protein tyrosine phosphatase inhibitory activity [5]. Insecticidal agents were also reported for penicianstinoid A, an austinoid-like meroterpenoid [6], as well as plant regulators for the dongtingnoids, diterpenoid glycosides which revealed promising seed-germination-promoting activities [7]. Accordingly, strains of *Penicillium* have gained considerable attention due to their ability to produce unusual secondary metabolites and have proved to be a prolific source of bioactive compounds. 

**Figure 1:**
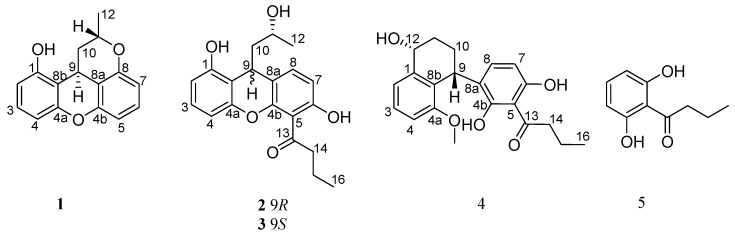
Chemical structures of compounds **1**–**5.**

In our search for new bioactive compounds from fungal sources [6,8,9], the fungus *Penicillium* sp. JY246 was isolated from the mangrove’s stem *Ceriops tagal*, collected from the South China Sea. The EtOAc extract of the fermentation broth showed significant activity against newly hatched larvae of *Helicoverpa armigera* Hubner. Chemical investigation of the fungus fermentation’s organic extract resulted in the isolation of four new xanthene derivatives (**1**–**4**), along with one known compound **5** (Figure 1). Herein, the isolation, structure elucidation, and insecticidal activity of these compounds are described.

## 2. Results and Discussion

### 2.1. Structure Elucidation

**Figure 2:**
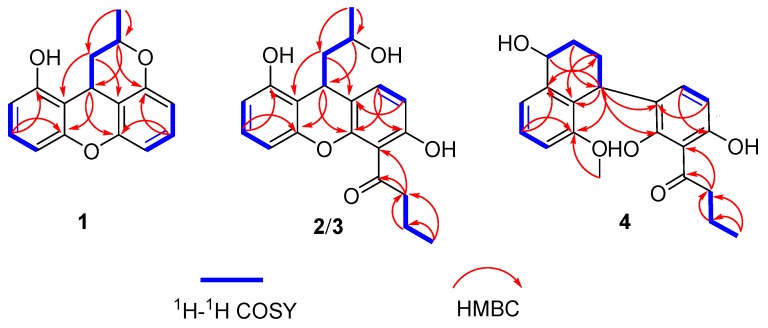
^1^H-^1^H COSY correlations and key HMBC correlations for compounds **1**–**4**.

Compound **1** was obtained as a white amorphous powder, and its molecular formula was determined to be C_16_H_14_O_3_ based on HRESIMS (Figure S8), accounting for 10 degrees of unsaturation. Analysis of the ^1^H NMR spectrum (Table 1, Figure S1) displayed six olefinic proton signals at *δ*_H_ 7.05 (dd, *J* = 8.4, 8.4 Hz, H-6), 7.04 (dd, *J* = 8.0, 8.0 Hz, H-3), 6.58 (dd, *J* = 8.0, 0.8 Hz, H-4), 6.55 (dd, *J* = 8.0, 0.8 Hz, H-2), 6.52 (dd, *J* = 8.4, 1.2 Hz, H-5), and 6.50 (dd, *J* = 8.4, 1.2 Hz, H-7); two methine signals at *δ*_H_ 4.64 (m, H-11) and 3.99 (dd, *J* = 12.4, 4.0 Hz, H-9); one methylene group at *δ*_H_ 3.17 (ddd, *J* = 13.2, 4.0, 1.6 Hz, H-10b) and 1.79 (dt, *J* = 12.4, 6.4 Hz, H-10a); and one methyl signal at *δ*_H_ 1.39 (d, *J* = 6.4 Hz, H-12). Interpretation of the ^13^C NMR and DEPT spectra (Table 2, Figures S2 and S3) revealed 16 carbon signals, attributable to twelve sp^2^-hybridized carbons (*δ*_C_ 156.9, 154.3, 152.3, 150.1, 127.9, 127.8, 110.4, 110.2, 109.3, 109.1, 107.0 and 106.5) indicating the presence of two benzene rings, two sp^3^ methine groups (*δ*_C_ 71.7 and 22.3), one sp^3^ methylene group (*δ*_C_ 33.0), and one methyl group (*δ*_C_ 20.7). The detailed structure of **1** was identified by interpretation of the COSY (Figure S6) and HMBC spectra (Figure 2 and Figure S5). The key HMBC correlations from H-9 to C-4a/C-4b, and further—H-10b to C-8a/C-8b, H-11 to C-8/C-9, and H_3_-12 to C-10/C-11—indicate that C-9 linked the two benzene rings at C-8a/C-8b, and the HMBC correlations from H-3 to C-4a and H-6 to C-4b indicated the presence of one oxygen bridge between C-4a and C-4b. So, the two benzene rings were connected through C-9 and oxygen-bridge to build up the skeleton of 9-methylene-9*H*-xanthene [10]. Furthermore, C-11 linked to C-8 through the oxygen bridge was deduced by chemical shift at C-8 (*δ*_C_ 154.3) and C-11 (*δ*_C_ 71.7). Hereto, the planar structure of **1** was elucidated (Figure 1).

**Figure 3:**
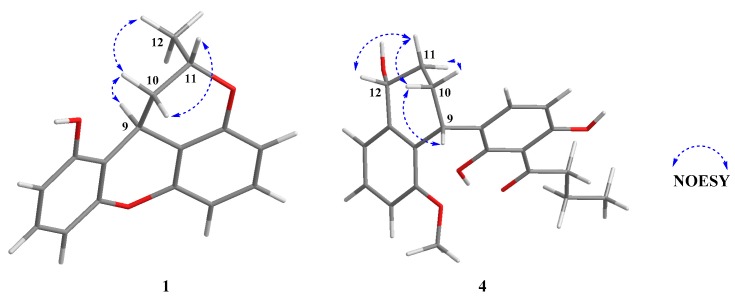
Energy-minimized 3D models of **1** and **4** with selected NOESY correlations.

**Figure 4:**
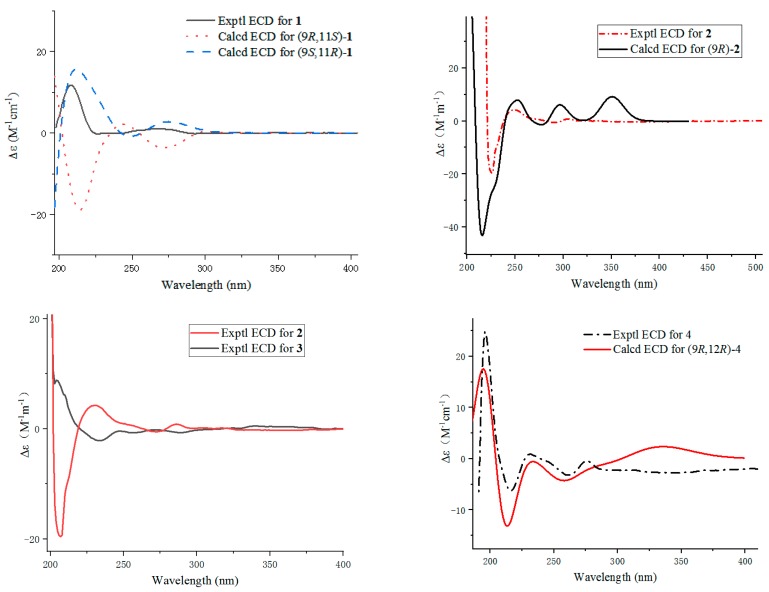
Experimental and calculated electronic circular dichroism (ECD) spectra of compounds **1**–**4**.

The relative configuration of **1** was revealed by the NOESY experiment (Figure 3 and Figure S7). The NOESY correlations of H-9 to H_3_-12 indicated that H-9 and H_3_-12 were on the contrary side of the H-11. The absolute configuration of **1** was determined by comparing experimental and calculated electronic circular dichroism (ECD) spectra for the truncated model (9*R*, 11*S*)-**1** and the truncated model (9*S*, 11*R*)-**1** using time-dependent density-functional theory (TDDFT). The DFT reoptimization of the initial Merck molecular force field (MMFF) minima was performed at the B3LYP/6 − 31 + *g* (d, p) level with a conductor-like polarizable continuum model (CPCM) solvent model for MeOH [11]. The theoretical spectrum of **1** showed the same Cotton effect with the experimental plot recorded in MeOH (Figure 4), which supported that the absolute configuration was 9*S*, 11*R*. Thus, the completed structure of **1** was elucidated as depicted in Figure 1, and was named penicixanthene A. 

Compound **2** was isolated as a colorless amorphous powder. The molecular formula of **2** was determined to be C_20_H_22_O_5_ (10 degrees of unsaturation) by HRESIMS (Figure S16). The ^1^H and ^13^C NMR data (Table 1 and Table 2, Figures S9 and S10) revealed that **2** belongs to the 9-methylene-9*H*-xanthene class [10], and suggested a close structural relationship to **1**. The obvious differences in ^1^H NMR spectrum were the disappearance of two aromatic protons signals at *δ*_H_ 6.50/H-5 and *δ*_H_ 7.05/H-6 in **2**. In addition, in the ^13^C NMR spectra, the C-5/C-6 signals moved downfield *δ*_C_ 110.2/127.9 in **1** vs*. δ*_C_ 110.7/157.3 in **2**, indicating that the disappearance of two aromatic protons were respectively replaced by a butan-1-one unit at C-5 and a hydroxy group at C-6 in **2**, which was further supported by HMBC (Figure S13). The existence of the butan-1-one unit was confirmed by HMBC correlations from H-14 to C-5/C-13/C-16, H-15 to C-13/C-14, and H-16 to C-14/C-15.

**Figure 5:**
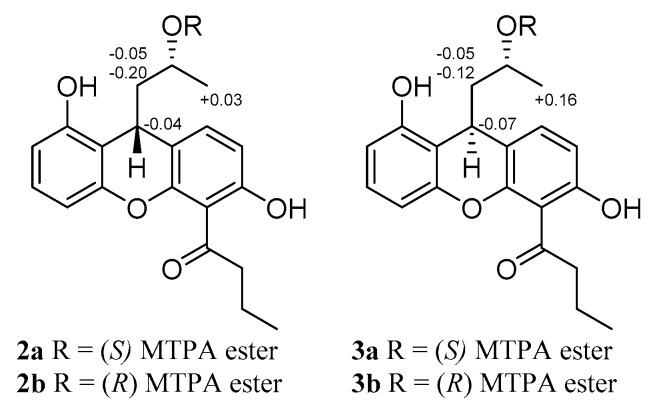
Reactions of compounds **2** and **3** with Mosher esters.

Furthermore, the oxygen-bridge between C-11 to C-8 in **1** was broken in **2**. The C-8 signal moved high-field significantly at *δ*_C_ 134.4 in **2** vs. *δ*_C_ 154.3 in **1** in the ^13^C NMR spectrum, and the H-8 signal at *δ*_H_ 6.69 in **2** in the ^1^H NMR spectrum, indicating that oxygen function at C-8 in **1** was replaced by an aromatic proton in **2**, which was confirmed by HMBC correlations (Figure 2) of H-7 to C-5/C-8a, H-8 to C-4b/C-6/C-9, H-9 to C-4a/C-4b, H-10 to C-8a/C-8b, H-11 to C-9, and H_3_-12 to C-10/C-11. The ^1^H-^1^H COSY (Figure S14) and HMBC spectra allowed the complete assignment of **2**. The absolute configuration of C-9 in **2** was resolved by comparing experimental and calculated ECD spectra using TDDFT (Figure 4) [11]. The absolute configuration of C-11 was determined by making MTPA esters of **2** [12], and the differences in ^1^H NMR (Figures S33 and S35) chemical shifts between (*S*)- and (*R*)-MTPA esters (Δ*δ* = *δ_S_* − *δ_R_*) (Figure 5) were calculated to assign the absolute configuration of C-11 to be *R*. Thus, the absolute configuration of **2** was established as 9*R*, 11*R*, and it was named penicixanthene B. 

Compound **3**, isolated as a colorless amorphous powder, was evidenced to have a molecular formula of C_20_H_22_O_5_ (10 degrees of unsaturation) from its HRESIMS data (Figure S24). The similarities in the NMR data (Figures S17 and S18) for **3** and **2** suggested that **3** was structurally similar to **2**, with the main differences being the chemical shifts of C-8b/C-9/C-10 (Table 2) and the coupling constants of H-9/H-10 (Table 1), and as indicated by the small coupling constant *J*_9,10_ = 1.2 Hz and 2D NMR data in **3** (vs. the coupling constant *J*_9,10_ = 8.0 Hz in **2**) [13], implying that the H-9 orientation of **3** was different from that of **2**. The absolute configuration of C-9 in **3** was established by comparison of experimental and calculated electronic circular dichroism (ECD) data [11]. The experimental spectrum of **3** showed the opposite Cotton effect with the experimental results of **2** recorded in MeOH (Figure 4), which supported that the absolute configuration of C-9 in **3** was 9*S*. The absolute configuration of C-11 was determined by making MTPA esters of **3** [12]. The differences in ^1^H NMR (Figures S37 and S39) chemical shifts between (*S*)- and (*R*)-MTPA esters (Δ*δ* = *δ_S_* − *δ_R_*) (Figure 5) were calculated to assign the absolute configuration of C-11 to be *R*. Thus, the absolute configuration of **3** was assigned as 9*S*, 11*R*, and the structure of **3** was defined as penicixanthene C.

Compound **4** was obtained as white amorphous powder, and the molecular formula was deduced to be C_21_H_24_O_5_ on the basis of HRESIMS (Figure S32), implying 10 degrees of unsaturation. Analysis of the ^1^H NMR spectrum (Table 1, Figure S25) displayed signals corresponding to five olefinic protons signal at *δ*_H_ 7.24 (dd, *J* = 8.0, 8.0 Hz, H-3), 7.06 (d, *J* = 8.0 Hz, H-4), 6.81 (d, *J* = 8.0 Hz, H-2), 6.22 (d, *J* = 8.4 Hz, H-8), and 6.05 (d, *J* = 8.4 Hz, H-7); two methine signals at *δ*_H_ 4.72 (m, H-12) and 4.63 (m, H-9); four methylene groups at *δ*_H_ 3.12 (t, *J* = 7.2 Hz, H-14), 2.22 (m, H-10b), 1.81 (m H-10a), 1.74 (m, H-11), and 1.72 (m, H-15); and two methyl groups at *δ*_H_ 3.57 (s, H-17) and 0.99 (t, *J* = 7.2 Hz, H-16). The ^13^C NMR spectrum (Table 2, Figure S26) displayed 21 signals, which were classified by DEPT (Figure S27) and HMQC spectra (Figure S28) as twelve sp^2^-hybridized carbons (*δ*_C_ 162.1, 159.7, 158.3, 141.6, 135.6, 128.9, 128.3, 125.4, 123.3, 110.9, 110.8, and 105.8) indicating the presence of two benzene rings, two sp^3^ methine groups (*δ*_C_ 67.9 and 32.3), four sp^3^ methylene groups (*δ*_C_ 47.2, 28.2, 23.9, and 19.2), and two methyl groups (*δ*_C_ 59.9 and 14.3). The above NMR spectroscopic data indicated the presence of a structure similar to nodulisporin F [13] with two subunits. One of the subunits was similar to **5** [14], isolated from the same source. The other subunit showed signals corresponding to the naphthalene part. The locations of connection between the two units at C-8a/C-9 were evidenced from HMBC correlations (Figure 2, Figure S29) of H-9 to C-4a/C-4b/C-8/C-8a/C-8b, as well as from H-8 to C-9. Based on the above data, the naphthalene part of **4** was linked to a 1-(2,6-dihydroxyphenyl)-butan-1-one moiety in para-position to the phenol group (at C-9). In addition, the C-4a and C-12 in **4** were replaced by a methoxyl group and a hydroxy group, respectively. 

The relative configuration of **4** was based on the NOESY (Figure S31) correlations, as indicated in Figure 3. The NOESY correlations of H-9 to H-10a, H-11a, and H-12, as well as H-10b to H-11b, indicated that H-9, H-10a, H-11a, and H-12 were on the contrary side of the H-10b and H-11b. The absolute configuration of **4** was elucidated as 9*R*, 12*R* by comparing its experimental ECD spectrum to the calculated spectrum (Figure 4) [11]. The structure of **4** was assigned, named as penicixanthene D.

### 2.2. Biological Activity

Compounds **1**–**5** were examined for insecticidal activity. In the test, we set up three parallel trials. Compounds **2** and **3** showed growth inhibition activities against newly hatched larvae of *Helicoverpa armigera* Hubner with the IC_50_ values 100 and 200 μg/mL, respectively. Azadirachtin was used as positive control with the IC_50_ value of 25 μg/mL. Compounds **1**, **3**, and **4** showed insecticidal activity against newly hatched larvae of *Culex quinquefasciatus* with LC_50_ values of 38.5 (±1.16), 11.6 (±0.58) and 20.5 (±1) μg/mL, respectively. Azadirachtin was used as positive control with the LC_50_ value of 8.8 (±0.58) μg/mL (Table 3).

## 3. Materials and Methods 

### 3.1. General Experimental Procedures

Optical rotations were measured on a JASCO P-1020 digital polarimeter (JASCO, Tokyo, Japan). IR spectra were recorded on a Thermo Nicolet 6700 (using KBr disks) spectrophotometer (Thermo, Madison, USA). 1D and 2D NMR spectra were measured on a Bruker AV-400 spectrometer with TMS as the internal standard (Bruker Corporation, Fällanden, Switzerland) (The PULPROG for testing NOESY was noesygpphpp, the temperature was 300 K, and the mix time of NOESY was 0.3 s). HRESIMS spectra were obtained on a Q-TOF Ultima Global GAA076 LC mass spectrometer (Waters, Milford, MA, USA). Preparative HPLC was used for an Agilent 1260 prep-HPLC system with an Agilent Eclipse XDB-C18 column (250 mm × 9.4 mm, 7 µm, Agilent Corporation, Santa Clara, CA, USA). The other experimental procedures were performed as reported previously [6]. 

### 3.2. Fungal Materials

The fungal strain *Penicillium* sp. JY246 was isolated from the stem of mangrove *Ceriops tagal*, collected in the South China Sea in July 2016. The fungus was identified according to its morphological characteristics and by comparison of the internal transcribed spacer (ITS) sequence amplification, primer pair ITS1 and ITS4 and sequencing of the ITS region. The sequence data has been submitted to GenBank with the accession number MK050979, and identified as *Penicillium*. 

The fungal strain was cultivated in 20 L potato glucose liquid medium (15 g of glucose and 30 g of sea salt in 1 L of potato infusion, in 1 L Erlenmeyer flasks each containing 300 mL of culture broth) at 25 °C without shaking for 4 weeks.

### 3.3. Extraction and Isolation

The fungal cultures were filtered through cheesecloth, and the filtrate was extracted with EtOAc (3 *×* 20 L, 24 h each). The organic extracts were concentrated in vacuo to yield an oily residue (23.6 g), which was subjected to silica gel column chromatography (CC) (petroleum ether, EtOAc *v/v*, gradient 100:0–0:100) to generate five fractions (Fr. 1–Fr. 5). Fraction Fr. 4 (5 g) was separated by silica gel CC and eluted with petroleum ether-EtOAc (from 3:1 to 0:1) to afford four subfractions (**4a**–**4d**). Subfraction **4b** were further separated by semi-preparative HPLC (MeOH–H_2_O, 45:55, *v*/*v*) to obtain **1** (6.0 mg), **4** (4.5 mg), and **5** (5.0 mg), and subfraction **4c** was further separated by semi-preparative HPLC with MeOH–H_2_O (35:65 *v*/*v*) to give **2** (3.3 mg) and **3** (3.5 mg).

*Penicixanthene* A (**1**)*:* white amorphous powder. [*α*]${}_{D}^{24}$ − 10.7 (*c* = 0.02, CHCl_3_). CD (*c* 2 × 10^-4^ mol/L, MeOH) *λ*_max_ (Δ*ε*) 211 (+11.8), 242 (−0.1), 269 (+1.1) nm; IR (KBr) *ν*_max_ 3405, 2923, 1747, 1740, 1230, 1064, 756 cm^-1^; ^1^H and ^13^C NMR see Table 1 and Table 2; HRESIMS *m/z* 255.1012 [M + H]^+^ (calcd. For C_16_H_15_O_3_, 255.1016).

*Penicixanthene* B (**2**)*:* white amorphous powder. [*α*]${}_{D}^{24}$ – 9.5 (*c* = 0.02, CHCl_3_). CD (*c* 2 × 10^-4^ mol/L, MeOH) *λ*_max_ (Δ*ε*) 207 (−19.6), 232 (+4.2), 252 (+0.8), 286 (+0.8) nm; IR (KBr) *ν*_max_ 3402, 2944, 1747, 1410, 1250, 1064, 818 cm^-1^; ^1^H and ^13^C NMR see Table 1 and Table 2; HRESIMS *m/z* 343.1537 [M + H]^+^ (calcd. for C_20_H_23_O_5_, 343.1540).

*Penicixanthene* C (**3**): white amorphous powder. [*α*]${}_{D}^{24}$ + 10.7 (*c* = 0.10, CHCl_3_). CD (*c* 2 × 10^-4^ mol/L, MeOH) *λ*_max_ (Δ*ε*) 207 (+8.3), 232 (−2.1), 252 (−0.7), 286 (−0.7) nm; IR (KBr) *ν*_max_ 3405, 2943, 1740, 1413, 1255, 1061, 820 cm^-1^; ^1^H and ^13^C NMR see Table 1 and Table 2; HRESIMS *m/z* 343.1537 [M + H]^+^ (calcd. for C_20_H_23_O_5_, 343.1540).

*Penicixanthene* D (**4**)*:* white amorphous powder. [*α*]${}_{D}^{24}$ + 30.7 (*c* = 0.10, CHCl_3_). CD (*c* 2 × 10^-4^ mol/L, MeOH) *λ*_max_ (Δ*ε*) 196 (+25.1), 215 (−5.2), 231 (+1.9), 276 (+0.5) nm; IR (KBr) *ν*_max_ 3265, 1631, 1237 cm^-1^; ^1^H and ^13^C NMR see Table 1 and Table 2; HRESIMS *m/z* 357.1700 [M + H]^+^ (calcd. for C_21_H_25_O_5_ 357.1697)

### 3.4. Preparation of (S)- and (R)-MTPA Ester Derivatives of Compounds **2** and **3**

Preparations of (*S*)- and (*R*)-MTPA ester derivatives of **2** and **3** were performed as described previously [6].

S-MTPA ester of **2** (**2a**): ^1^H NMR (CDCl_3_, 400 MHz) *δ*_H_: 7.23 (1H, d, *J* = 8.4 Hz, H-8), 7.15 (1H, t, *J* = 6.8 Hz, H-3), 6.96 (1H, dd, *J* = 8.8, 0.8 Hz, H-2), 6.81 (1H, d, *J* = 8.4 Hz, H-7), 6.46 (1H, dd, *J* = 8.8, 0.8 Hz, H-4), 5.34 (1H, m, H-11), 4.12 (1H, m, H-9), 3.59 (2H, m, H-14), 2.53 (1H, m, H-10b), 2.01 (2H, m, H-15), 1.74 (1H, m, H-10a), 1.17 (3H, d, *J* = 6.0 Hz, H-12), 0.77 (3H, t, *J* = 7.2 Hz, H-16). ESI-MS *m/z* 773.1 [M − H]^−^.

R-MTPA ester of **2** (**2b**): ^1^H NMR (CDCl_3_, 400 MHz) *δ*_H_: 7.29 (1H, m, H-8), 7.10 (1H, m, H-3), 6.99 (1H, m, H-2), 6.46 (1H, m, H-7), 6.23 (1H, m, H-4), 5.32 (1H, m, H-11), 4.08 (1H, m, H-9), 3.58 (2H, m, H-14), 2.33 (1H, m, H-10b), 1.99 (2H, m, H-15), 1.69 (1H, m, H-10a), 1.20 (3H, m, H-12), 0.72 (3H, m, H-16). ESI-MS *m/z* 773.1 [M − H]^−^.

S-MTPA ester of **3** (**3a**): ^1^H NMR (CDCl_3_, 400 MHz) *δ*_H_: 7.35 (1H, m, H-8), 7.30 (1H, m, H-3), 6.81 (1H, m, H-2), 6.69 (1H, m, H-7), 6.49 (1H, m, H-4), 5.34 (1H, m, H-11), 4.52 (1H, m, H-9), 3.58 (2H, m, H-14), 2.61 (1H, m, H-10b), 2.11 (2H, m, H-15), 2.01 (1H, m, H-10a), 1.30 (3H, d, *J* = 4.8 Hz, H-12), 0.74 (3H, t, *J* = 7.2 Hz, H-16). ESI-MS *m/z* 773.1 [M − H]^−^.

R-MTPA ester of **3** (**3b**): ^1^H NMR (CDCl_3_, 400 MHz) *δ*_H_: 7.30 (1H, m, H-8), 7.07 (1H, m, H-3), 6.96 (1H, m, H-2), 6.56 (1H, m, H-7), 6.36 (1H, m, H-4), 5.35 (1H, m, H-11), 4.59 (1H, m, H-9), 3.55 (2H, m, H-14), 2.56 (1H, m, H-10b), 2.10 (2H, m, H-15), 1.89 (1H, m, H-10a), 1.31 (3H, d, *J* = 6.0 Hz, H-12), 0.74 (3H, t, *J* = 6.4 Hz, H-16). ESI-MS *m/z* 773.1 [M − H]^−^.

### 3.5. Computational Section 

As previously reported [6,15,16].

### 3.6. Insecticidal Activities against Newly Hatched Larvae of Helicoverpa armigera Hubner

Insecticidal activity against newly hatched larvae of *H. armigera* Hubner was evaluated according to the previously reported methods [6]. Newly hatched larvae were raised under 25 ± 1 °C and a relative humidity of 80%. DMSO was used as the negative control, azadirachtin was used as the positive control, and artificial diet was used as the blank control. The number of dead larvae was recorded on the 2nd, 4th, 6th, and 8th day after treatment, respectively [17].

### 3.7. Insecticidal Activities against Newly Hatched Larvae of Culex quinquefasciatus

Insecticidal activity against newly hatched larvae of *C. quinquefasciatus* was evaluated according to the previously reported methods [17,18]. DMSO was used as the negative control, azadirachtin was used as the positive control, and 10 mL dechlorinated water was used as the blank control. The number of dead larvae was recorded on the 1st, 2nd, 3rd, and 4th day after treatment, respectively.

## 4. Conclusions

In summary, four new xanthene derivatives, penicixanthenes A–D (**1**–**4**), along with one known compound **5**, were obtained from the mangrove-derived fungus *Penicillium* sp. JY246. Furthermore, the absolute configurations of **2** and **3** were determined by modified Mosher’s method and calculated electronic circular dichroism data. Compounds **2** and **3** showed growth inhibition activity against newly hatched larvae of *H. armigera* Hubner with the IC_50_ values 100 and 200 μg/mL, respectively. Compounds **1**, **3**, and **4** showed insecticidal activity against newly hatched larvae of *C. quinquefasciatus* with LC_50_ values of 38.5 (±1.16), 11.6 (±0.58), and 20.5 (±1) μg/mL, respectively. 

## Figures and Tables

**Table 1:** ^1^H NMR data (400 MHz, *δ* in ppm, *J* in Hz) for **1**–**4**.

Position	1 *^a^*	2 *^b^*	3 *^b^*	4 *^b^*
2	6.55, dd (8.0, 0.8)	6.35, dd (8.0, 0.8)	6.35, dd (8.0, 0.8)	6.81, d (8.0)
3	7.04, dd (8.0, 8.0)	6.90, dd (8.0, 8.0)	6.94, dd (8.0, 8.0)	7.24, dd (8.0, 8.0)
4	6.58, dd (8.0, 0.8)	6.26, dd (8.0, 0.8)	6.29, dd (8.0, 0.8)	7.06, d (8.0)
5	6.52, dd (8.4, 1.2)			
6	7.05, dd (8.4, 8.4)			
7	6.50, dd (8.4, 1.2)	6.18, d (8.4)	6.16, d (8.4)	6.05, d (8.4)
8		6.69, d (8.4)	6.57, d (8.4)	6.22, d (8.4)
9	3.99, dd (12.4, 4.0)	4.52, dd (10.4, 8.0)	4.50, dd (7.6, 1.2)	4.63, m
10a	1.79, td (12.4, 6.4)	1.49, td (13.6, 10.4)	1.76, ddd (13.6, 7.6, 4.4)	1.81, m
10b	3.17, ddd (13.2, 4.0, 1.6)	2.44, ddd (13.6, 8.0, 1.6)	2.06, td (13.6, 1.2)	2.22, m
11	4.64, m	4.04, m	3.82, m	1.74, m
12	1.39, d (6.4)	1.28, d (6.4)	1.25, d (6.4)	4.72, m
14		3.11, t (7.2)	3.13, t (7.2)	3.12, t (7.2)
15		1.71, m	1.73, m	1.72, m
16		0.98, t (7.2)	0.99, t (7.2)	0.99, t (7.2)
17				3.57, s

*^a^* DMSO-*d*_6_ *^b^* CD_3_OD.

**Table 2:** ^13^C NMR data (100 MHz, *δ* in ppm) for **1**–**4**.

Position	1 *^a^*	2 *^b^*	3 *^b^*	4 *^b^*
1	156.9, C	159.7, C	159.9, C	141.6, C
2	107.0, CH	109.3, CH	108.7, CH	110.8, CH
3	127.8, CH	128.2, CH	128.6, CH	128.3, CH
4	110.4, CH	108.7, CH	107.4, CH	123.3, CH
4a	152.3, C	157.3, C	158.3, C	158.3, C
4b	150.1, C	161,9, C	161,9, C	162.1, C
5	110.2, CH	110.7, C	110.9, C	110.9, C
6	127.9, CH	157.3, C	159.9, C	159.7, C
7	106.5, CH	107.0, CH	106.0, CH	105.8, CH
8	154.3, C	134.4, CH	136.6, CH	135.6, CH
8a	109.3, C	126.2, C	125.3, C	125.4, C
8b	109.1, C	114.3, C	111.9, C	128.9, C
9	22.3, CH	31.8, CH	30.5, CH	32.3, CH
10	33.0, CH_2_	40.4, CH_2_	36.1, CH_2_	23.9, CH_2_
11	71.7, CH	73.4, CH	68.7, CH	28.2, CH_2_
12	20.7, CH_3_	21.6, CH_3_	21.7, CH_3_	67.9, CH
13		209.7, C	209.8, C	209.9, C
14		47.6, CH_2_	47.6, CH_2_	47.2, CH_2_
15		19.1, CH_2_	19.1, CH_2_	19.2, CH_2_
16		14.3, CH_3_	14.3, CH_3_	14.3, CH_3_
17				59.9, OCH_3_

*^a^* DMSO-*d*_6_ *^b^* CD_3_OD.

**Table 3:** Biological activities of **1**–**5**.

Compounds	IC_50_ (µg/mL)	LC_50_ (µg/mL)
	*Helicoverpa armigera* Hubner	*Culex quinquefasciatus*
**1**	>200	38.5 (±1.16)
**2**	100	>80
**3**	200	11.6 (±0.58)
**4**	>200	23.5 (±1)
**5**	>200	>80
Azadirachtin *^a^*	25	8.8 (±0.58)

*^a^* Azadirachtin was used as a positive control.

## Supplementary Materials

The following are available online at https://www.mdpi.com/1660-3397/17/12/649/s1, ^1^H NMR, ^13^C NMR, DEPT, HMQC, COSY, HMBC, NOESY, and HRESIMS spectra of compounds **1**–**4**. Figure S1: ^1^H NMR (DMSO-*d*_6_, 400 MHz) spectrum of **1**. Figure S2: ^13^C NMR (DMSO-*d*_6_, 100 MHz) spectrum of **1**. Figure S3: DEPT (DMSO-*d*_6_, 100 MHz) spectrum of **1**. Figure S4: HMQC spectrum of **1**. Figure S5: HMBC spectrum of **1**. Figure S6: ^1^H-^1^H COSY spectrum of **1**. Figure S7: NOESY spectrum of **1**. Figure S8: HRESIMS spectrum of **1**. Figure S9: ^1^H NMR (CD_3_OD, 400 MHz) spectrum of **2**. Figure S10: ^13^C NMR (CD_3_OD, 100 MHz) spectrum of **2**. Figure S11: DEPT (CD_3_OD, 100 MHz) spectrum of **2**. Figure S12: HMQC spectrum of **2**. Figure S13: HMBC spectrum of **2**. Figure S14: ^1^H-^1^H COSY spectrum of **2**. Figure S15: NOESY spectrum of **2**. Figure S16: HRESIMS spectrum of **2**. Figure S17: ^1^H NMR (CD_3_OD, 400 MHz) spectrum of **3**. Figure S18: ^13^C NMR (CD_3_OD, 100 MHz) spectrum of **3**. Figure S19: DEPT (CD_3_OD, 100 MHz) spectrum of **3**. Figure S20: HMQC spectrum of **3**. Figure S21: HMBC spectrum of **3**. Figure S22: ^1^H-^1^H COSY spectrum of **3**. Figure S23: NOESY spectrum of **3**. Figure S24: HRESIMS spectrum of **3**. Figure S25: ^1^H NMR (CD_3_OD, 400 MHz) spectrum of **4**. Figure S26: ^13^C NMR (CD_3_OD, 100 MHz) spectrum of **4**. Figure S27: DEPT (CD_3_OD, 100 MHz) spectrum of **4**. Figure S28: HMQC spectrum of **4**. Figure S29: HMBC spectrum of **4**. Figure S30: COSY spectrum of **4**. Figure S31: NOESY spectrum of **4**. Figure S32: HRESIMS spectrum of **4**. Figure S33: ^1^H NMR (CDCl_3_, 400 MHz) of *S*-MTPA ester of **2a**. Figure S34: ESIMS spectrum of **2a**. **Figure S35**: ^1^H NMR (CDCl_3_, 400 MHz) of *R*-MTPA ester of **2b**. Figure S36: ESIMS spectrum of **2b**. **Figure S37**: ^1^H NMR (CDCl_3_, 400 MHz) of *S*-MTPA ester of **3a.** Figure S38: ESIMS spectrum of **3a**. **Figure S39**: ^1^H NMR (CDCl_3_, 400 MHz) of *R*-MTPA ester of **3b**. Figure S40: ESIMS spectrum of **3b**.

## References

[1] Tang J.W., Kong L.M., Zu W.Y., Hu K., Li X.N., Yang B.C., Wang W.G., Sun H.D., Li Y., Puno P.T. (2019). Isopenicins A-C: Two types of antitumor meroterpenoids from the plant endophytic fungus *Penicillium* sp. sh18. Org. Lett..

[2] Liu S., Su M.Z., Song S.J., Hong J.K., Chung H.Y., Jung J.H. (2018). An anti-inflammatory PPAR-γ agonist from the jellyfish-derived fungus *Penicillium chrysogenum* J08NF-4. J. Nat. Prod..

[3] Liu S., Su M.Z., Song S.J., Jung J.H. (2017). Marine-derived *Penicillium* species as producers of cytotoxic metabolites. Mar. Drugs.

[4] Ariantari N.P., Ancheeva E., Wang C.Y., Mándi A., Knedel T.O., Kurtán T., Chaidir C., Müller W.E.G., Kassack M.U., Janiak C. (2019). Indole diterpenoids from an endophytic *Penicillium* sp.. J. Nat. Prod..

[5] Kong F.D., Fan P., Zhou L.M., Ma Q.Y., Xie Q.Y., Zheng H.Z., Zheng Z.H., Zhang R.S., Yuan J.Z., Dai H.F. (2019). Penerpenes A-D, Four indole terpenoids with potent protein tyrosine phosphatase inhibitory activity from the marine-derived fungus *Penicillium* sp. KFD28. Org. Lett..

[6] Bai M., Zheng C.J., Huang G.L., Mei R.Q., Wang B., Luo Y.P., Zheng C., Niu Z.G., Chen G.Y. (2019). Bioactive meroterpenoids and isocoumarins from the mangrove-derived fungus *Penicillium* sp. TGM112. J. Nat. Prod..

[7] Bie Q., Chen C.M., Yu M.Y., Guo J.R., Wang J.P., Liu J.J., Zhou Y., Zhu H.C., Zhang Y.H. (2019). Dongtingnoids A-G: fusicoccane diterpenoids from a *Penicillium* species. J. Nat. Prod..

[8] Huang G.L., Zhou X.M., Bai M., Liu Y.X., Zhao Y.L., Luo Y.P., Niu Y.Y., Zheng C.J., Chen G.Y. (2016). Dihydroisocoumarins from the mangrove-derived fungus *Penicillium citrinum*. Mar. Drugs.

[9] Bai M., Huang G.L., Mei R.Q., Wang B., Luo Y.P., Nong X.H., Chen G.Y., Zheng C.J. (2019). Bioactive lactones from the mangrove-derived fungus *Penicillium* sp. TGM112. Mar. Drugs.

[10] Cao J.Q., Yao Y., Chen H., Qiao L., Zhou Y.Z., Pei Y.H. (2007). A New xanthene from *Blumea riparia*. Chin. Chem. Lett..

[11] Choukchou-Braham N., Asakawa Y., Lepoittevin J.P. (1994). Isolation, structure determination and synthesis of new dihydroisocoumarins from *Ginkgo biloba* L.. Tetrahedron Lett..

[12] Kusumi T., Fujita Y., Ohtani I., Kakisawa H. (1991). Anomaly in the modified Mosher’s method: absolute configurations of some marine cembranolides. Tetrahedron Lett..

[13] Dai J.Q., Krohn K., Draeger S., Schulz B. (2009). New naphthalene-chroman coupling products from the endophytic fungus, *Nodulisporium* sp. from Erica arborea. Eur. J. Org. Chem..

[14] Yang L.J., Liao H.X., Bai M., Huang G.L., Luo Y.P., Niu Y.Y., Zheng C.J., Chen G.Y. (2018). One new cytochalasin metabolite isolated from a mangrovederived fungus *Daldinia eschscholtzii* HJ001. Nat. Prod. Res..

[15] Elnaggar M.S., Ebrahim W., Mándi A., Kurtán T., Müller W.E.G., Kalscheuer R., Singab A., Lin W.H., Liu Z., Proksch P. (2017). Hydroquinone derivatives from the marine-derived fungus *Gliomastix* sp.. RSC Adv..

[16] Ren J., Ding S.S., Zhu A., Cao F., Zhu H.J. (2017). Bioactive azaphilone derivatives from the fungus *Talaromyces aculeatus*. J. Nat. Prod..

[17] Guo Z.K., Gai C.J., Cai C.H., Chen L.L., Liu S.B., Zeng Y.B., Yuan J.Z., Mei W.L., Dai H.F. (2017). Metabolites with insecticidal activity from *Aspergillus fumigatus* JRJ111048 isolated from mangrove plant *Acrostichum specioum* endemic to hainan island. Mar. Drugs.

[18] Zhang W.F., Crickmore N., George Z., Xie L., He Y.Q., Li Y.Z., Tang J.L., Tian L., Wang X., Fang X.J. (2012). Characterization of new highly mosquitocidal isolate of *Bacillus thuringiensis*—an alternative to *Bti*?. J. Invertebr. Pathol..

