# Supplementary material for: Four New Insecticidal Xanthene Derivatives from the Mangrove-Derived Fungus Penicillium sp. JY246
Source: Mar Drugs. 2019 Nov 20;17(12):649. doi: 10.3390/md17120649 (PMC6950184; doi:10.3390/md17120649)
Supplement: Supplementary file 1 [file marinedrugs-17-00649-s001.pdf]

Supplementary Material

Four New Insecticidal Xanthene Derivatives from the Mangrove-Derived Fungus *Penicillium* sp. JY246

Meng Bai [1,2,†,] Cai-Juan Zheng [1,2,†], Xu-Hua Nong [1,2], Xue-Ming Zhou [1,2], You-Ping Luo [1,2], Guang-Ying Chen [1,2,*]

[1] Key Laboratory of Tropical Medicinal Resource Chemistry of Ministry of Education, Hainan Normal University, Haikou, Hainan 571127, China; xxbai2014@163.com (M.B.); caijuan2002@163.com (C.-J.Z.); nongxuhua4883@163.com (X.-H.N.); xueming2009211@126.com (X.-M.Z.); dengpengfei@gmail.com (Y.-P. L)

[2] Key Laboratory of Tropical Medicinal Plant Chemistry of Hainan Province, College of Chemistry and Chemical Engineering, Hainan Normal University, Haikou, Hainan 571127, China; xxbai2014@163.com (M.B.); caijuan2002@163.com (C.-J.Z.); nongxuhua4883@163.com (X.-H.N.); xueming2009211@126.com (X.-M.Z.); dengpengfei@gmail.com (Y.-P. L)

* Correspondence: chgying123@163.com (G.-Y.C.); Tel.: +86-898-6588-9422; Fax: +86-898-6588-9422

† These authors contributed equally to this work.

List of Supporting Information

Figure S1. ^{1}H NMR (DMSO-d_6, 400 MHz) spectrum of **1**.

Figure S2 ^{13}C NMR (DMSO-d_6, 100MHz) spectrum of **1**.

Figure S3 DEPT (DMSO-d_6, 100 MHz) spectrum of **1**.

Figure S4 HMQC spectrum of **1**.

Figure S5 HMBC spectrum of **1**.

Figure S6 COSY spectrum of **1**.

Figure S7 NOESY spectrum of **1**.

Figure S8 HRESIMS spectrum of **1**.

Figure S9 ^{1}H NMR (CD_3OD, 400 MHz) spectrum of **2**.

Figure S10 ^{13}C NMR (CD_3OD, 100 MHz) spectrum of **2**.

Figure S11 DEPT (CD_3OD, 100 MHz) spectrum of **2**.

Figure S12 HMQC spectrum of **2**.

Figure S13 HMBC spectrum of **2**.

Figure S14 COSY spectrum of **2**.

Figure S15 NOESY spectrum of **2**.

Figure S16 HRESIMS spectrum of **2**.

Figure S17 ^{1}H NMR (CD_3OD, 400 MHz) spectrum of **3**.

Figure S18 ^{13}C NMR (CD_3OD, 100 MHz) spectrum of **3**.

Figure S19 DEPT (CD_3OD, 100 MHz) spectrum of **3**.

Figure S20 HMQC spectrum of **3**.

Figure S21 HMBC spectrum of **3**.

Figure S22 COSY spectrum of **3**.

Figure S23 NOESY spectrum of **3**.

Figure S24 HRESIMS spectrum of **3**.

Figure S25. ^{1}H NMR (CD_3OD, 400 MHz) spectrum of **4**.

Figure S26. ^{13}C NMR (CD_3OD, 100 MHz) spectrum of **4**.

Figure S27. DEPT (CD_3OD, 100 MHz) spectrum of **4**.

Figure S28. HMQC spectrum of **4**.

Figure S29. HMBC spectrum of **4**.

Figure S30. COSY spectrum of **4**.

Figure S31. NOESY spectrum of **4**.

Figure S32. HRESIMS spectrum of **4**.

Figure S33. ^{1}H NMR ($CDCl_3$, 400 MHz) of *S*-MTPA ester of **2a**

Figure S34. ESIMS spectrum of **2a**

Figure S35. ^{1}H NMR ($CDCl_3$, 400 MHz) of *R*-MTPA ester of **2b**

Figure S36. ESIMS spectrum of **2b**

Figure S37. ^{1}H NMR ($CDCl_3$, 400 MHz) of *S*-MTPA ester of **3a**

Figure S38. ESIMS spectrum of **3a**

Figure S39. ^{1}H NMR ($CDCl_3$, 400 MHz) of *R*-MTPA ester of **3b**

Figure S40. ESIMS spectrum of **3b**

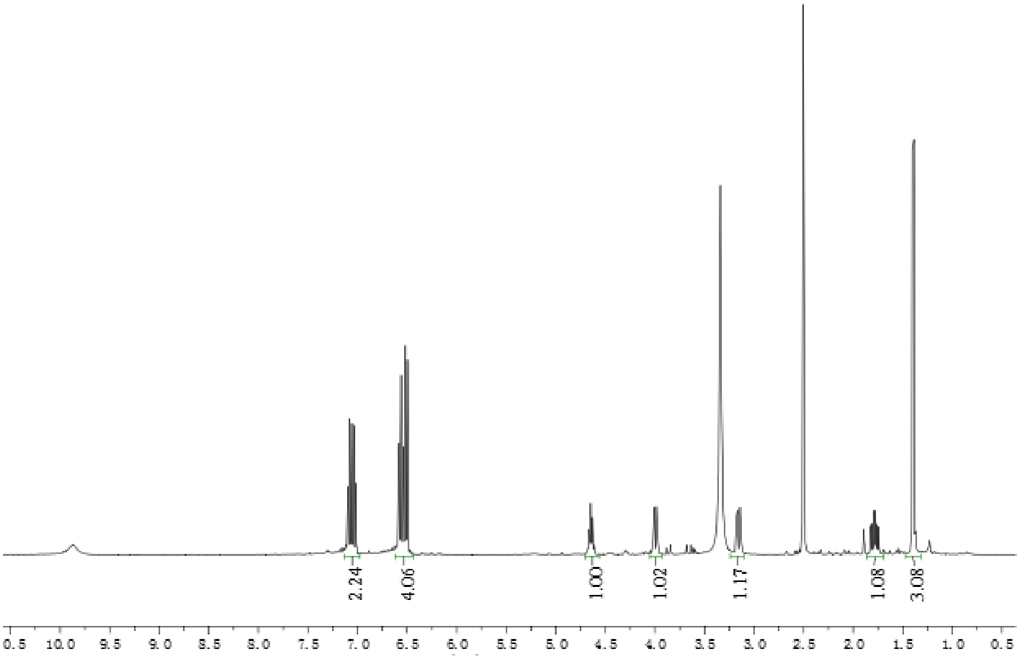

Figure S1. ^{1}H NMR (DMSO-d_6, 400 MHz) spectrum of **1**.

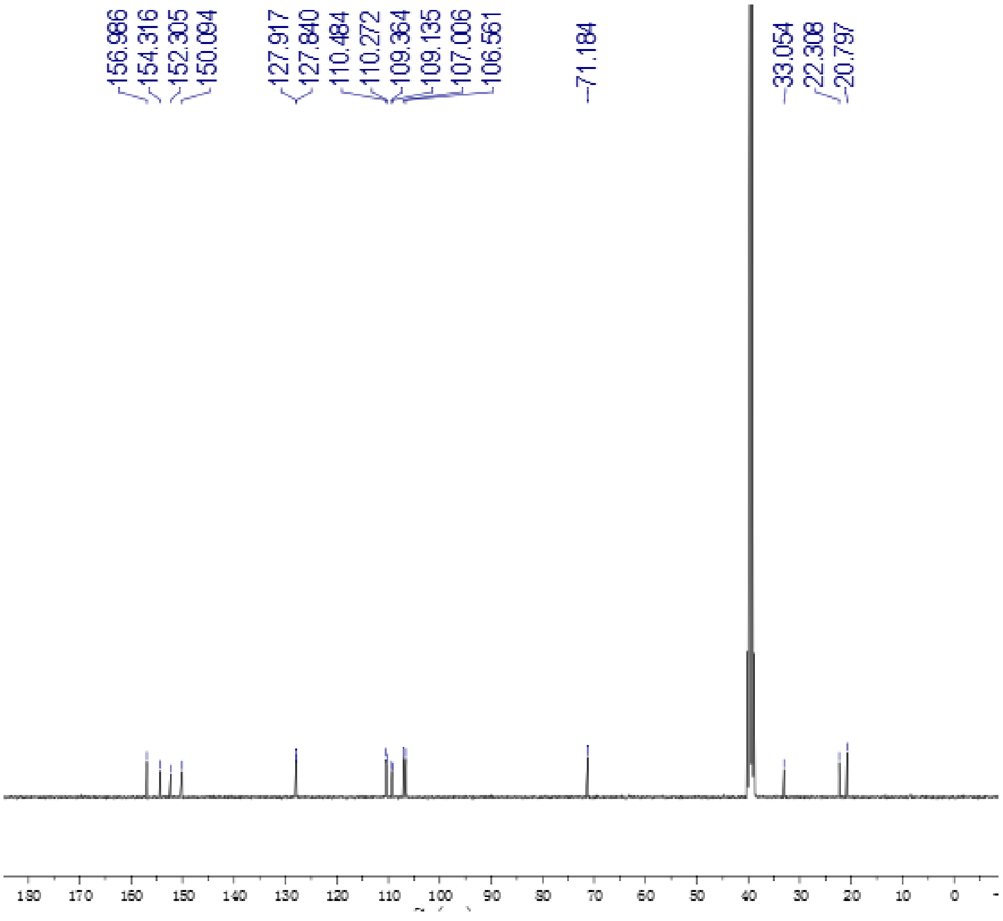

Figure S2 ^{13}C NMR (DMSO-d_6, 100MHz) spectrum of **1**.

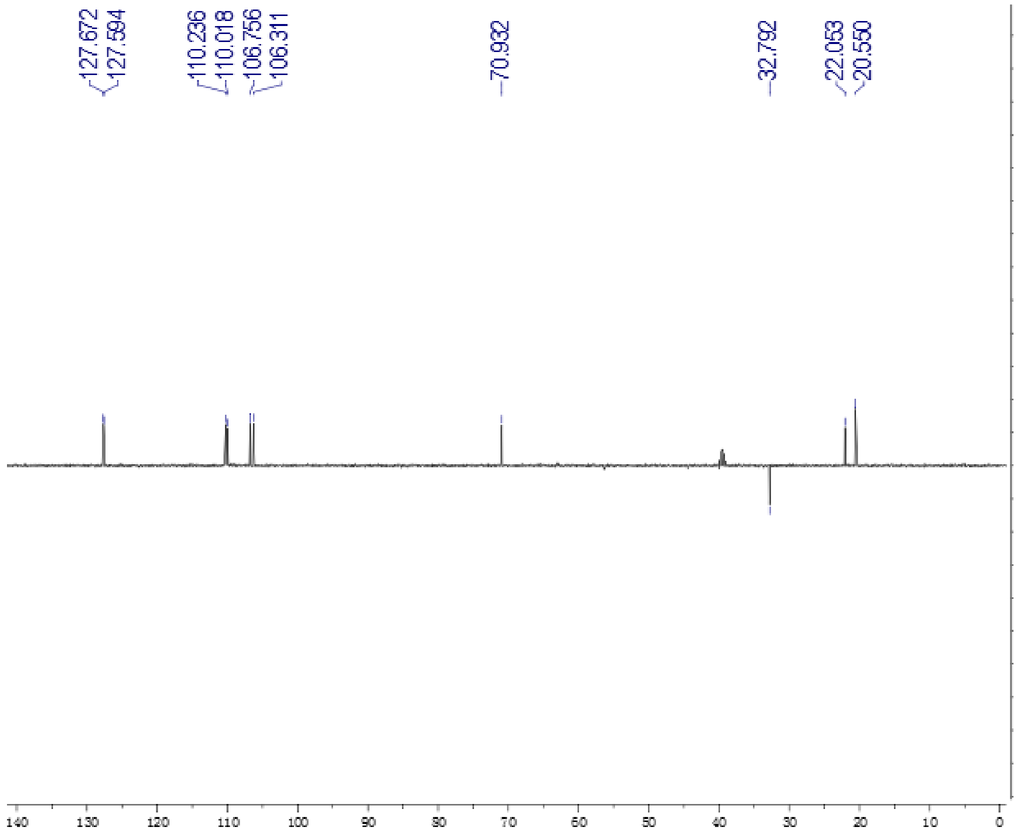

Figure S3 DEPT (DMSO-d_6, 100 MHz) spectrum of **1**.

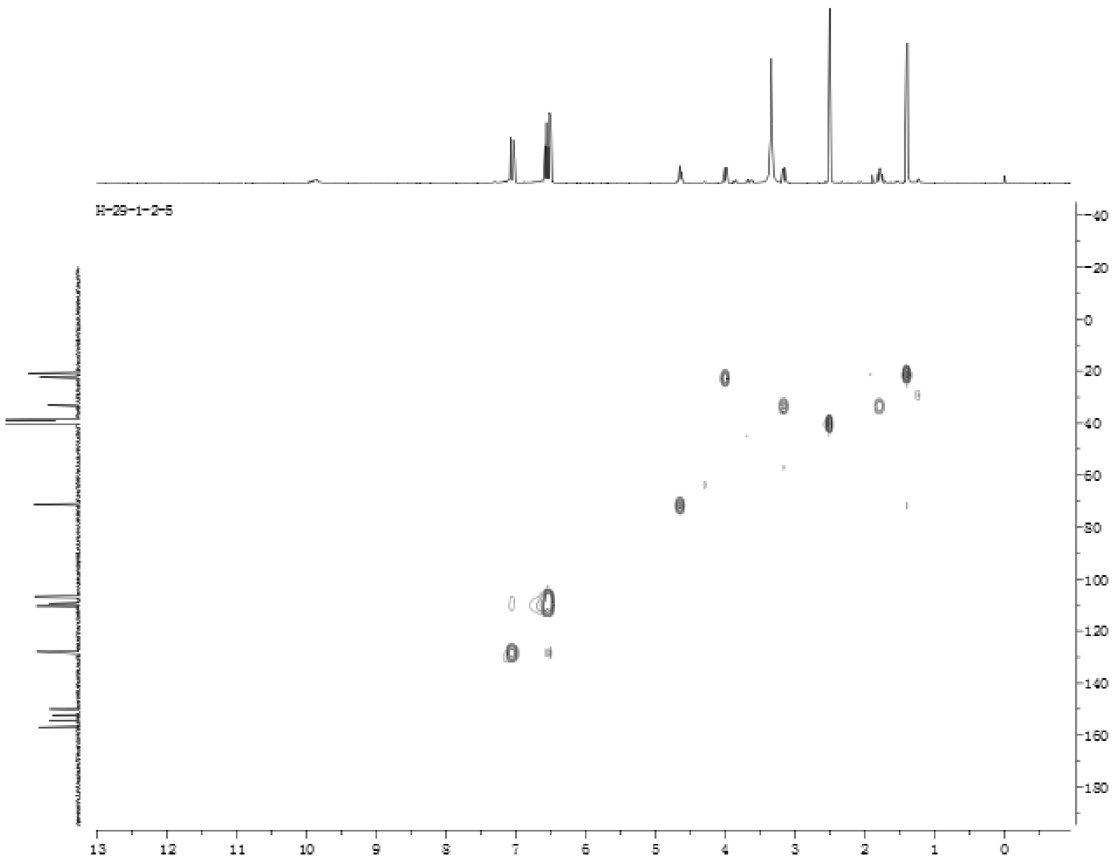

Figure S4 HMQC spectrum of **1**.

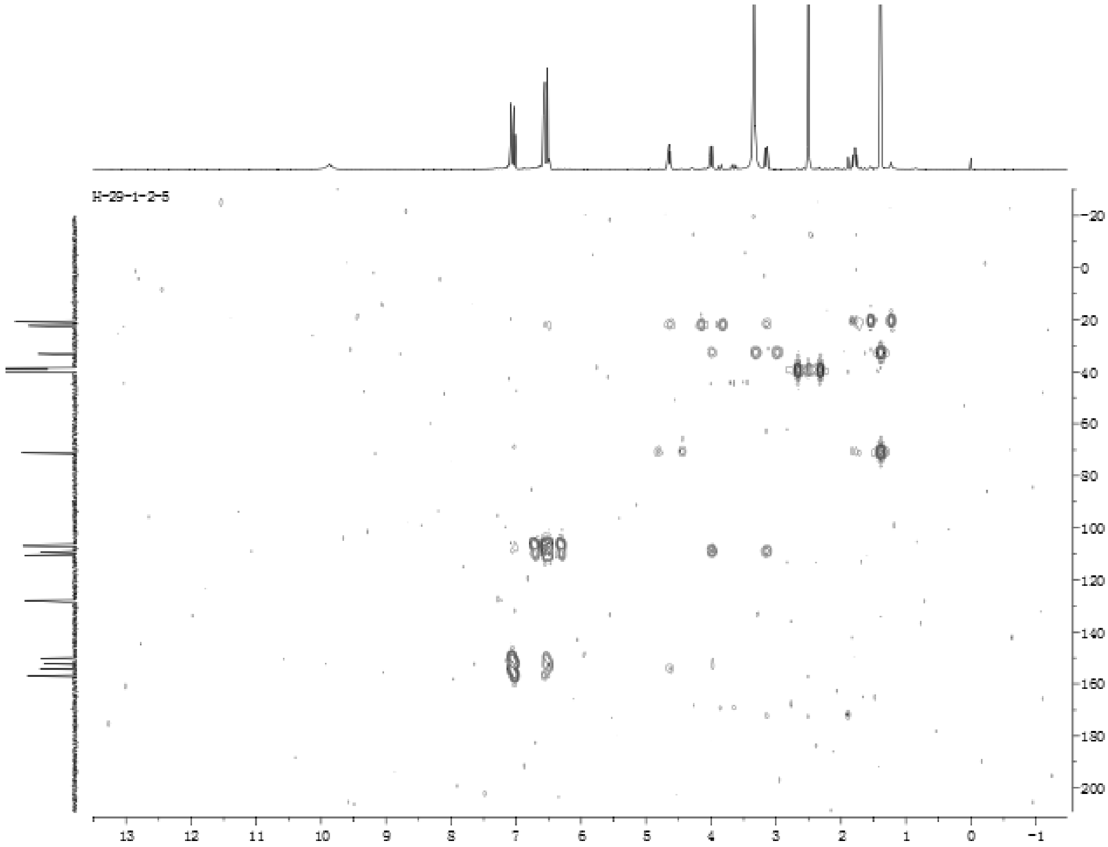

Figure S5 HMBC spectrum of **1**.

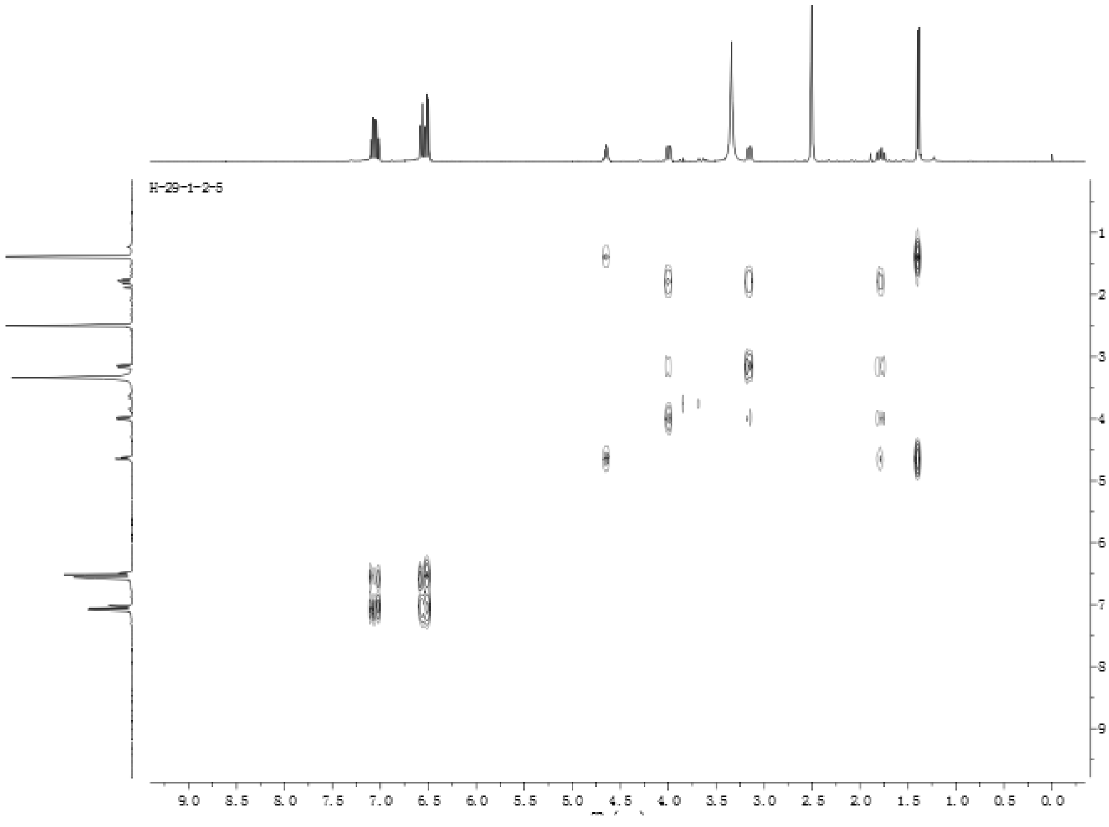

Figure S6 COSY spectrum of **1**.

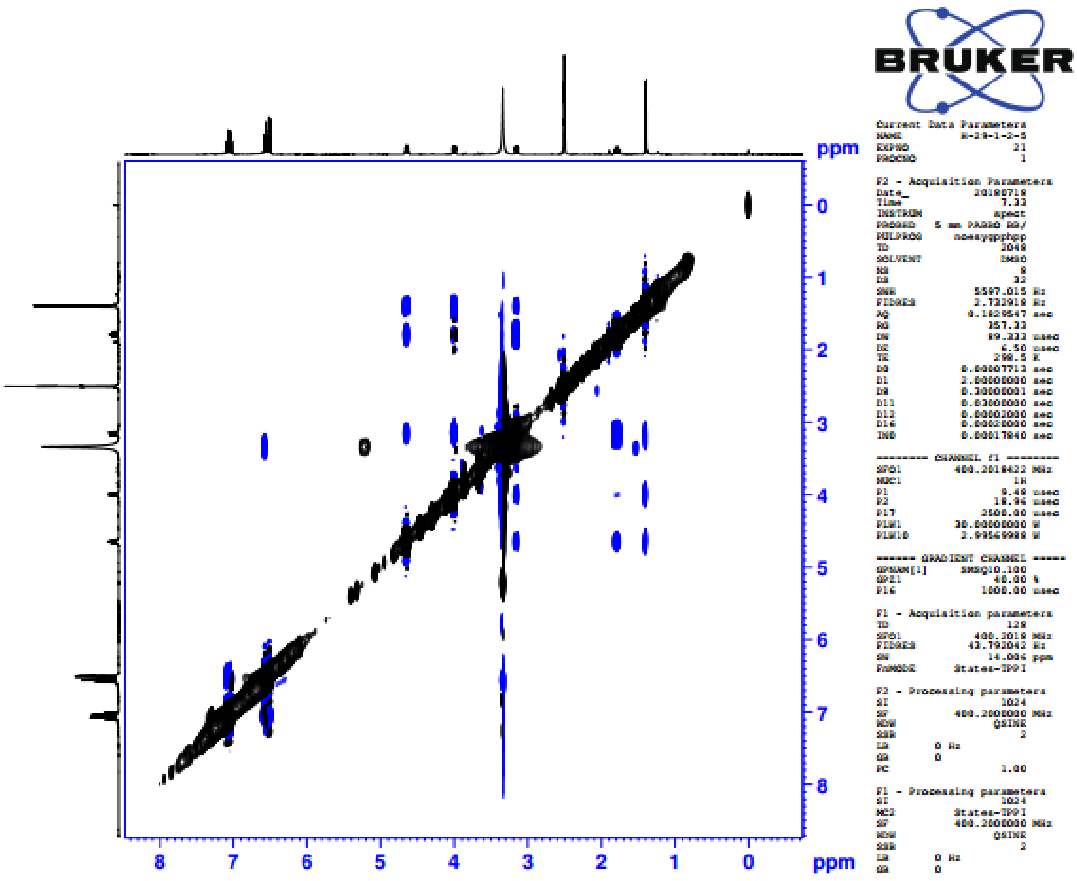

Figure S7 NOESY spectrum of **1**.

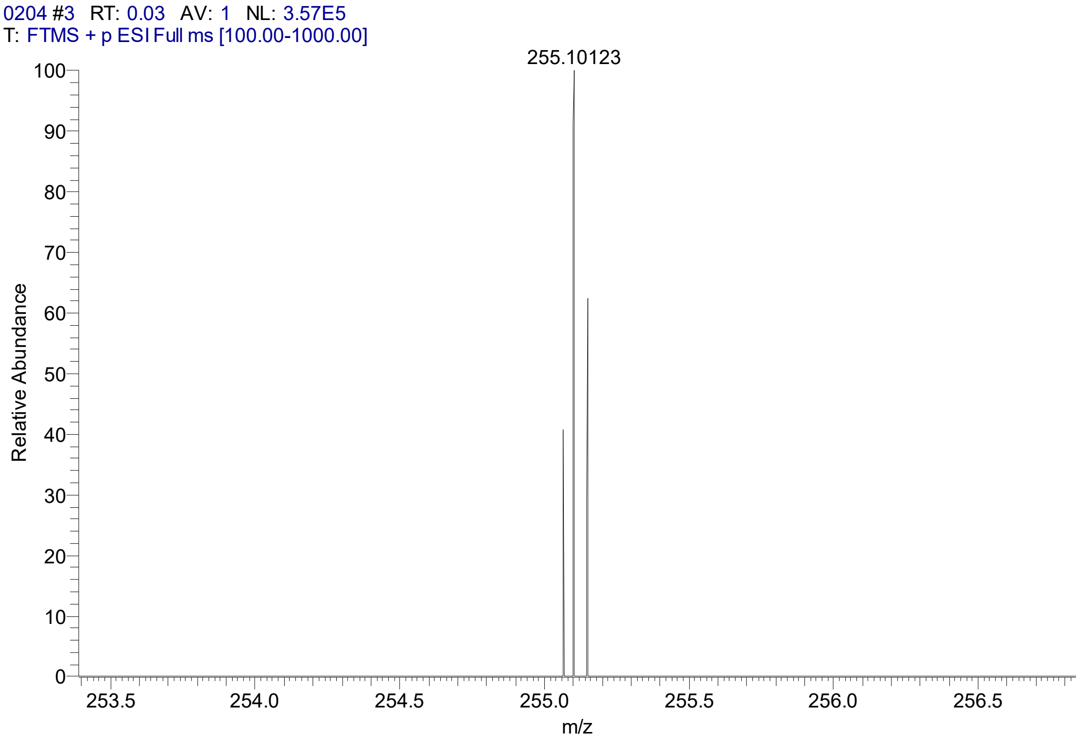

Figure S8 HRESIMS spectrum of **1**.

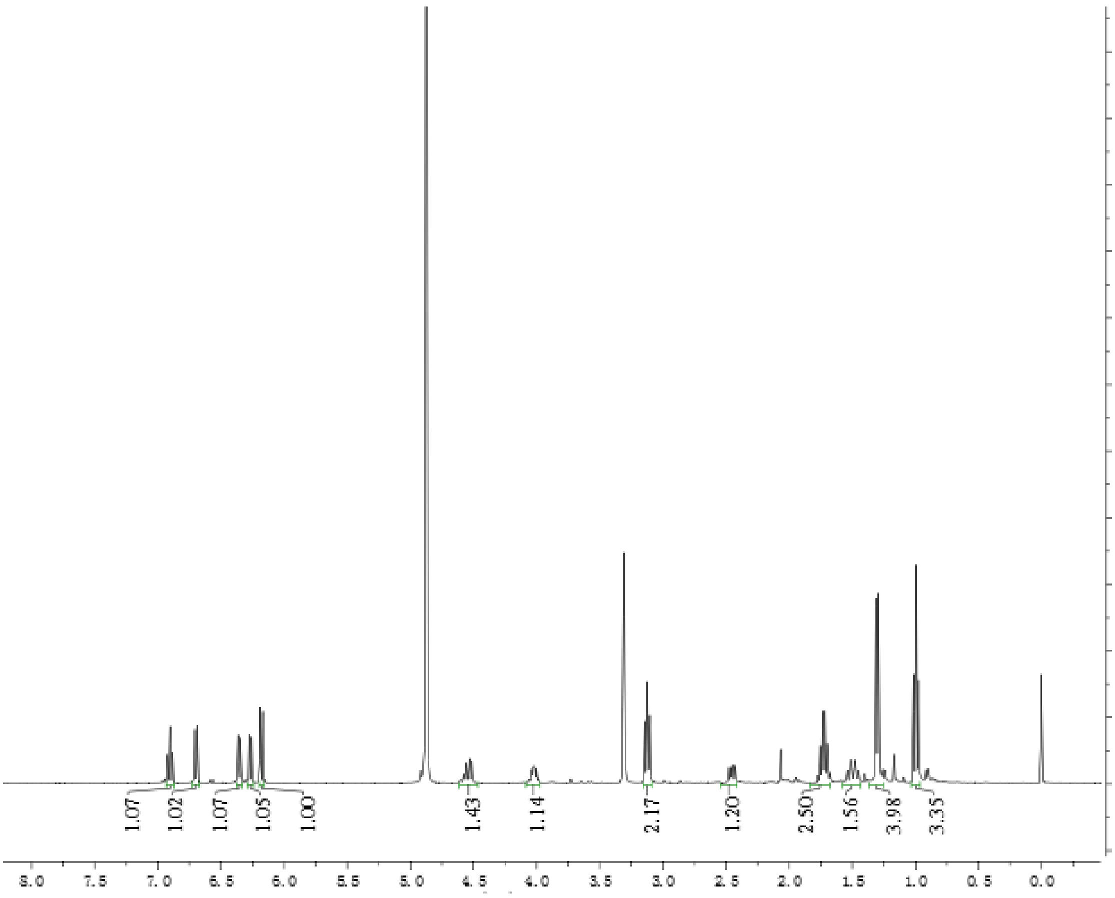

Figure S9 ¹H NMR (CD_3OD, 400 MHz) spectrum of **2**.

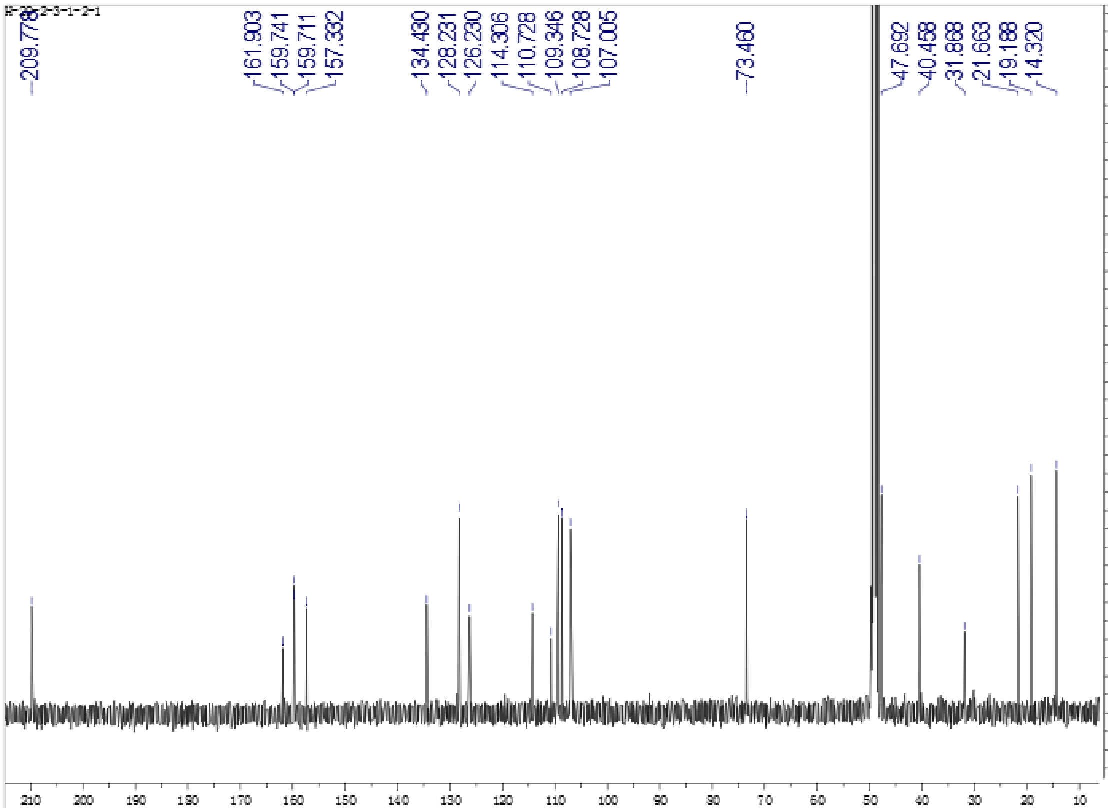

Figure S10 ¹³C NMR (CD_3OD, 100 MHz) spectrum of **2**.

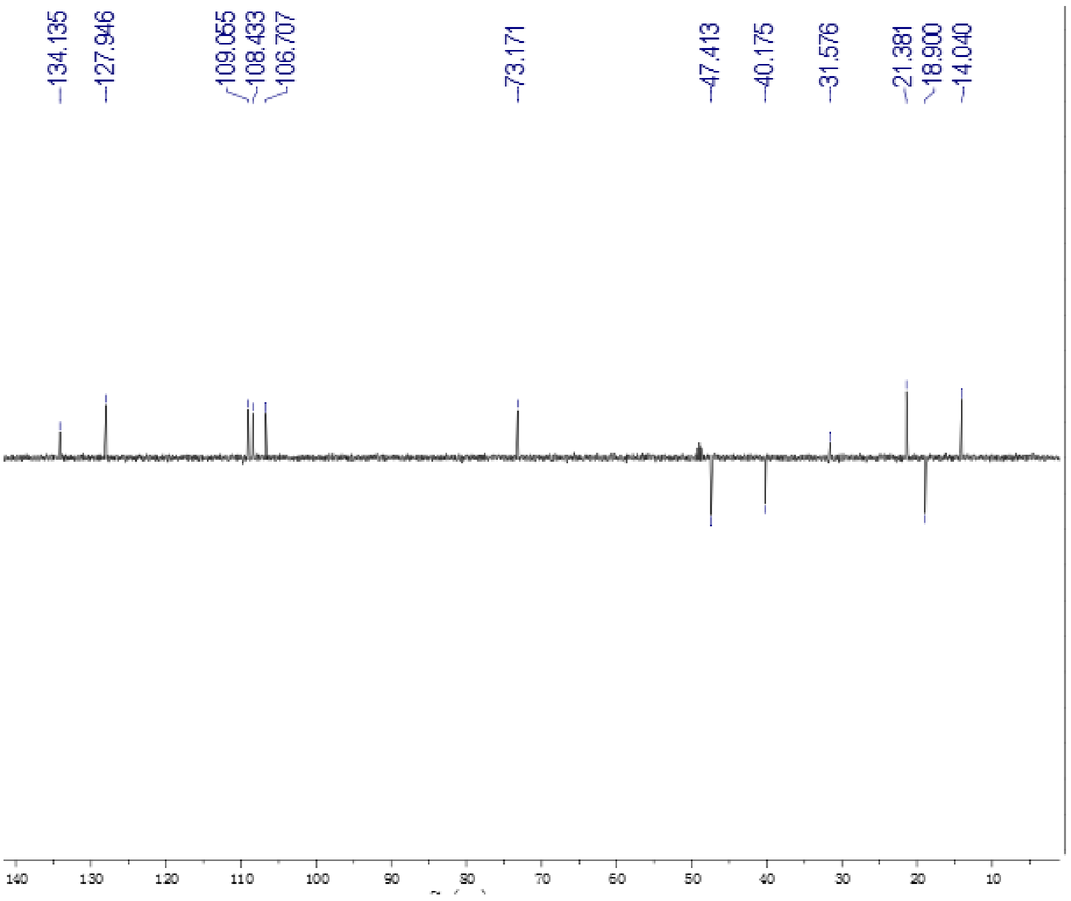

Figure S11 DEPT (CD_3OD, 100 MHz) spectrum of **2**.

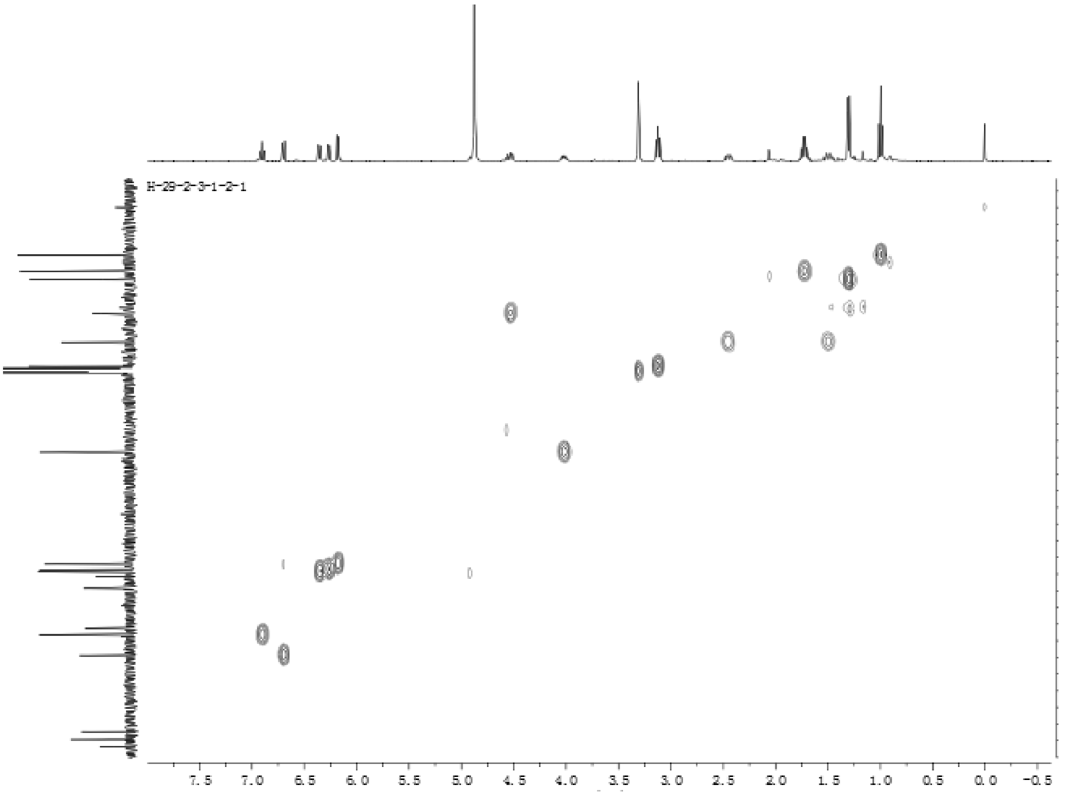

Figure S12 HMQC spectrum of **2**.

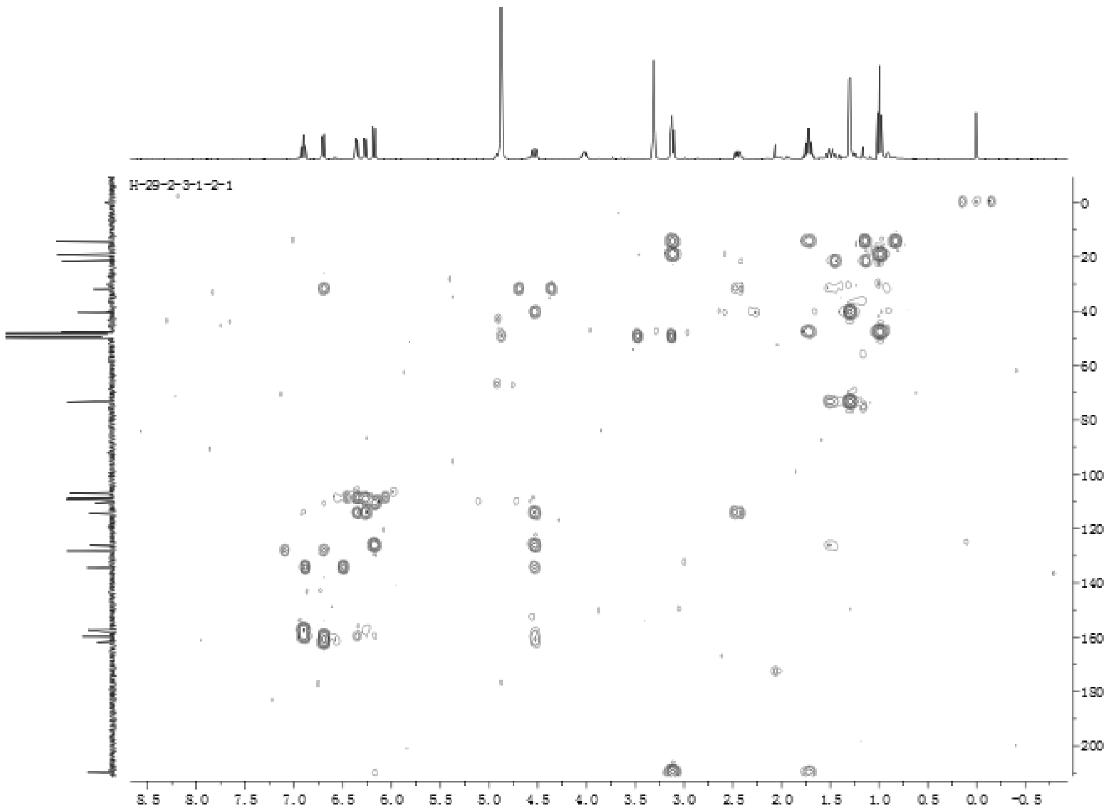

Figure S13 HMBC spectrum of **2**.

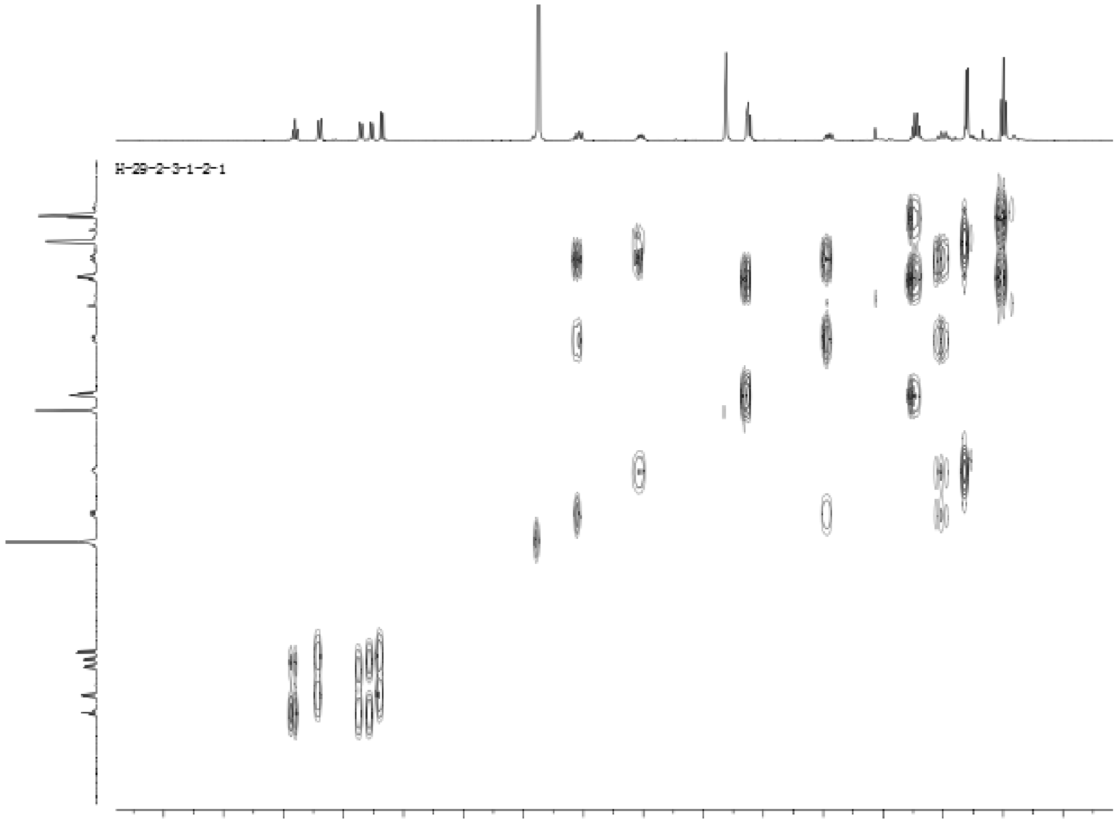

Figure S14 COSY spectrum of **2**.

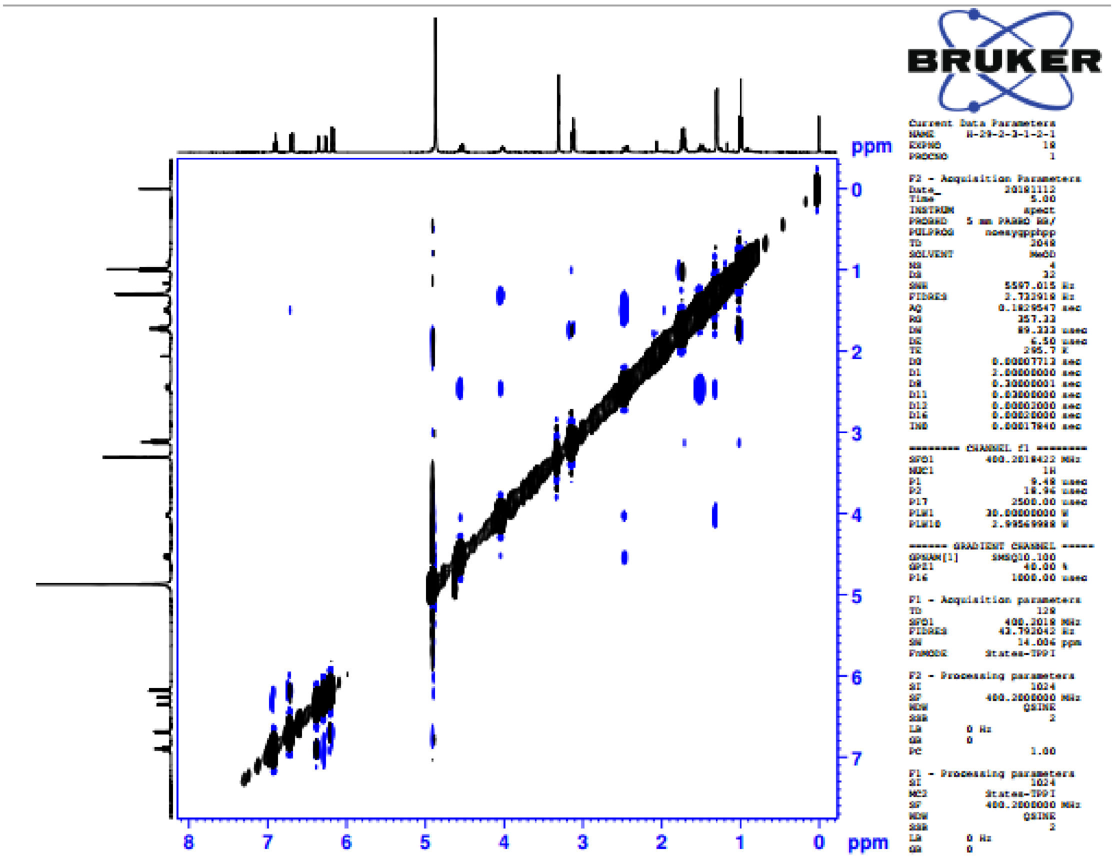

Figure S15 NOESY spectrum of **2**.

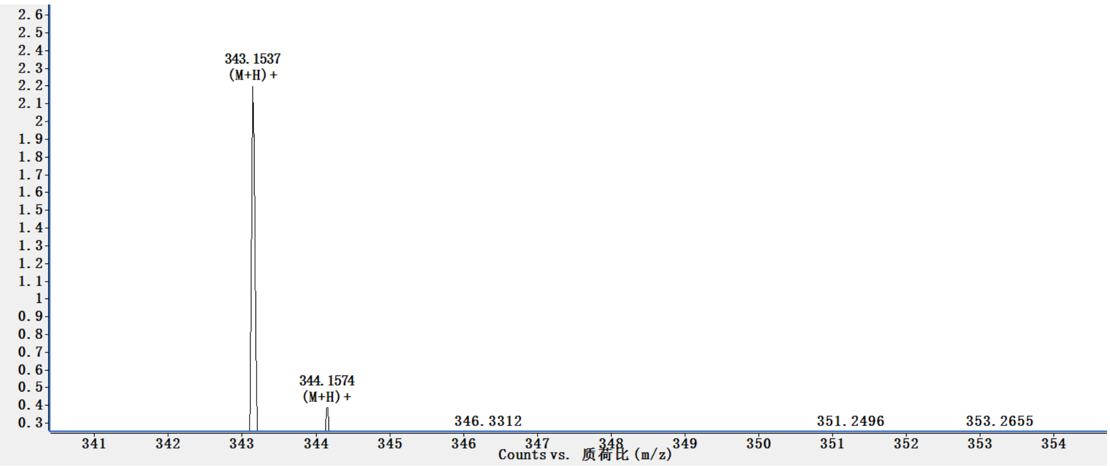

Figure S16 HRESIMS spectrum of **2**.

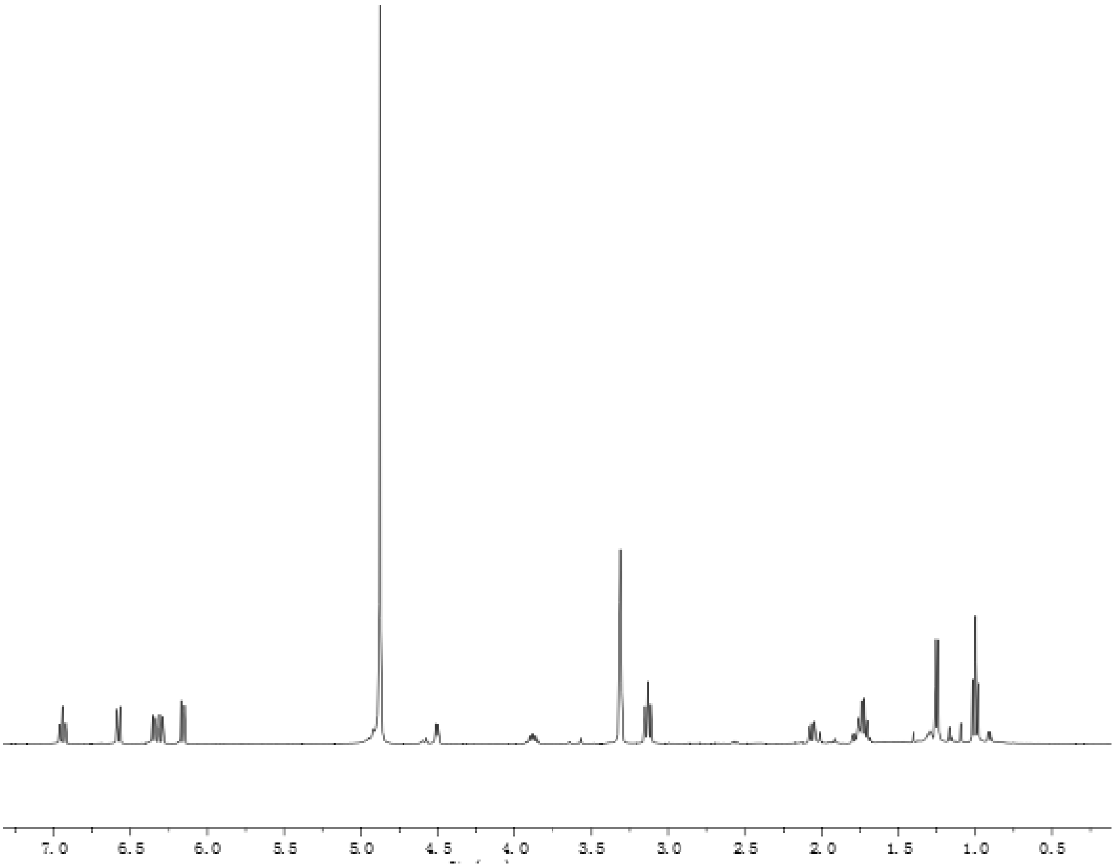

Figure S17 ^{1}H NMR (CD_3OD, 400 MHz) spectrum of **3**.

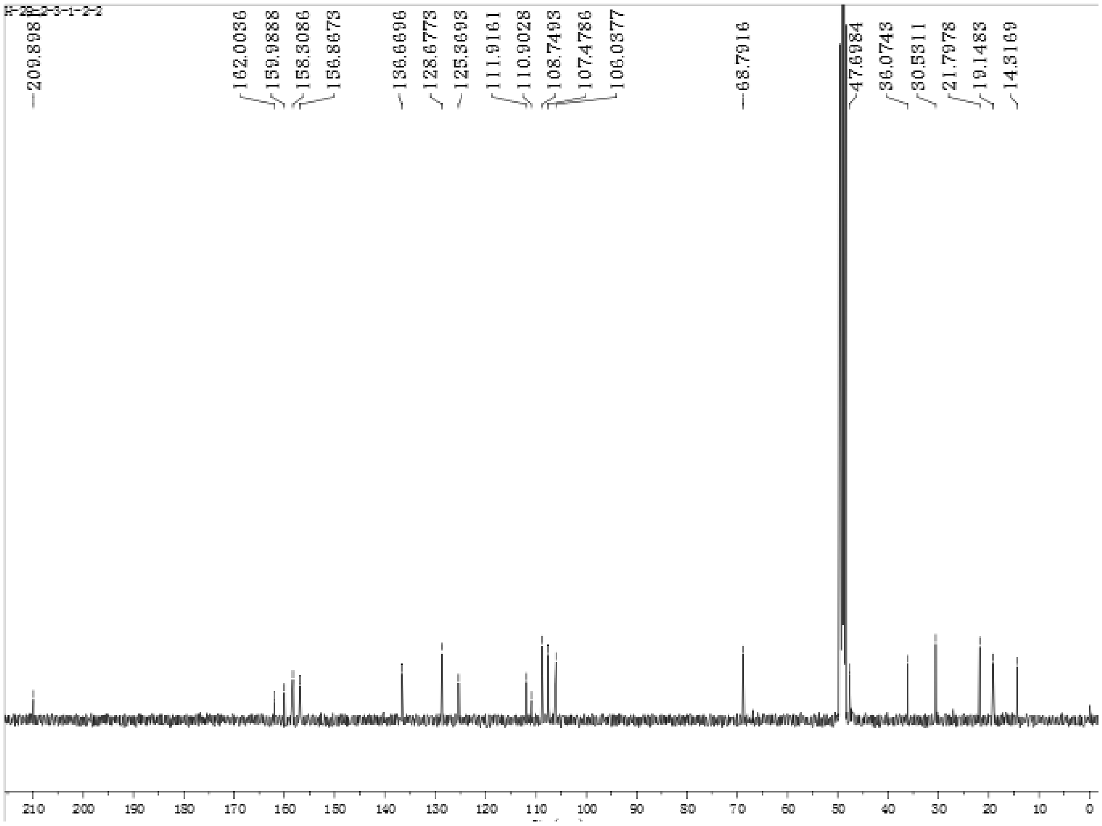

Figure S18 ^{13}C NMR (CD_3OD, 100 MHz) spectrum of **3**.

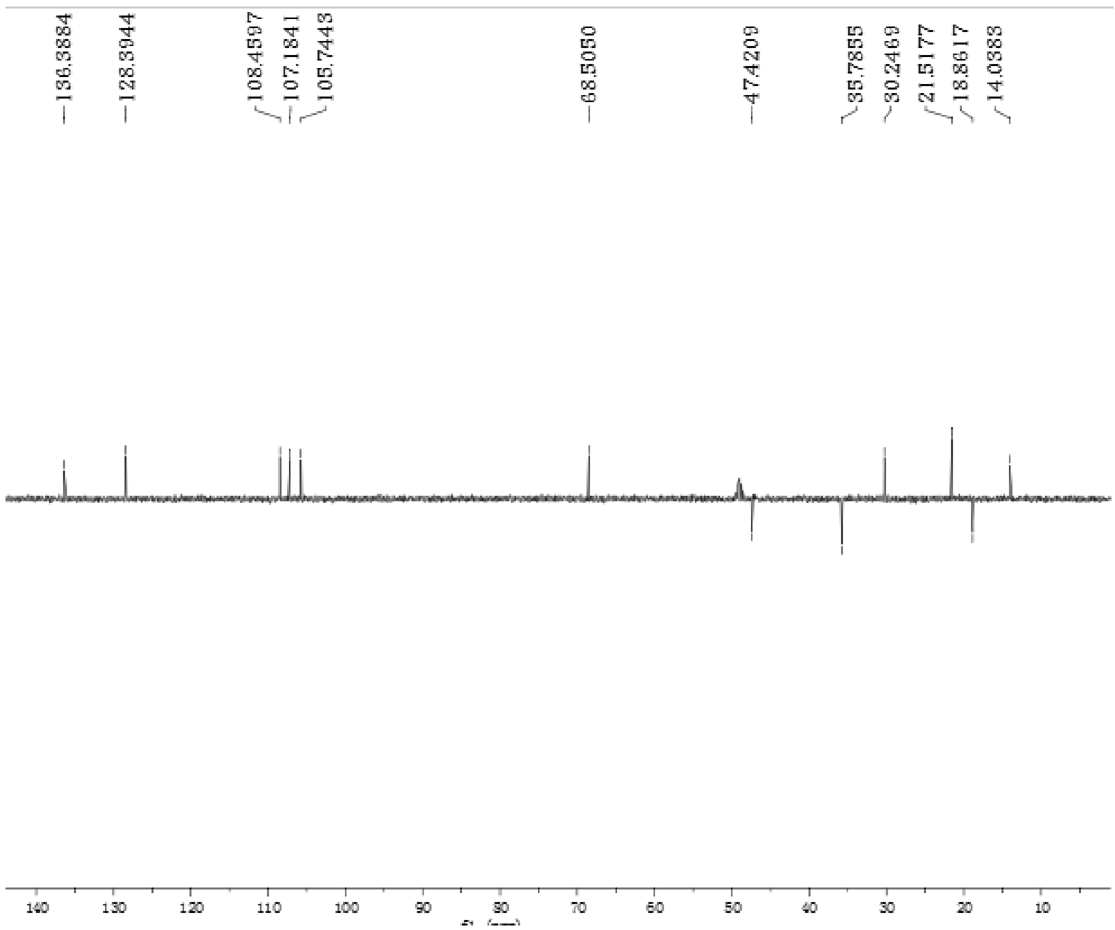

Figure S19 DEPT (CD_3OD, 100 MHz) spectrum of **3**.

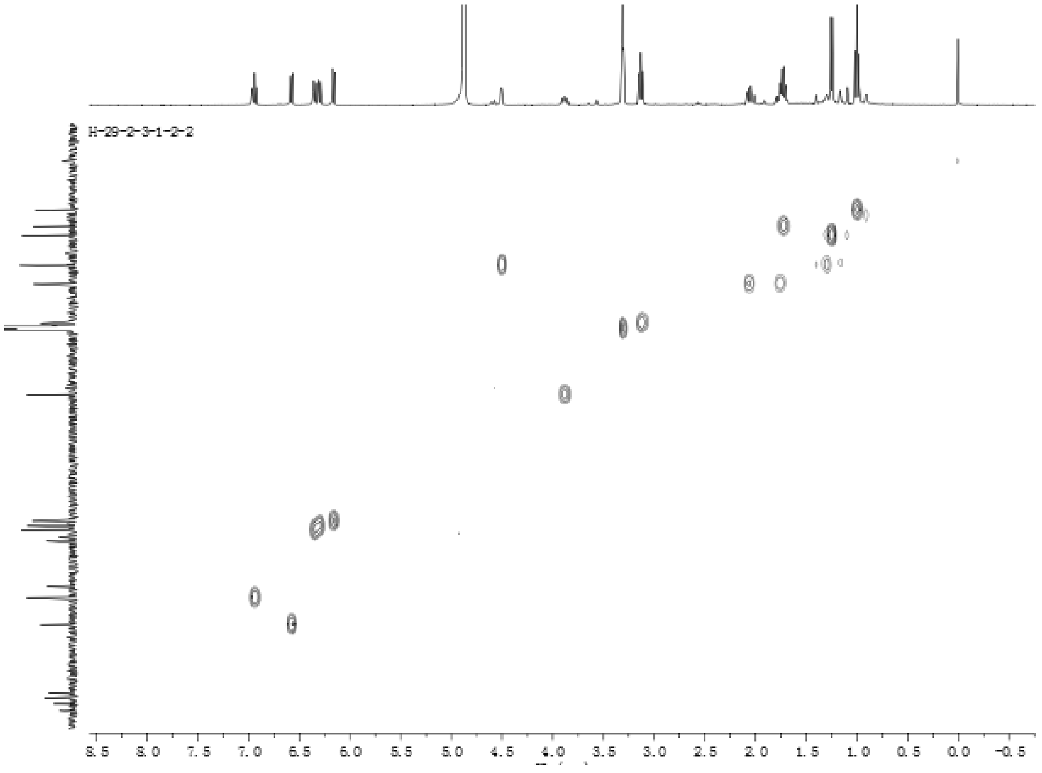

Figure S20 HMQC spectrum of **3**.

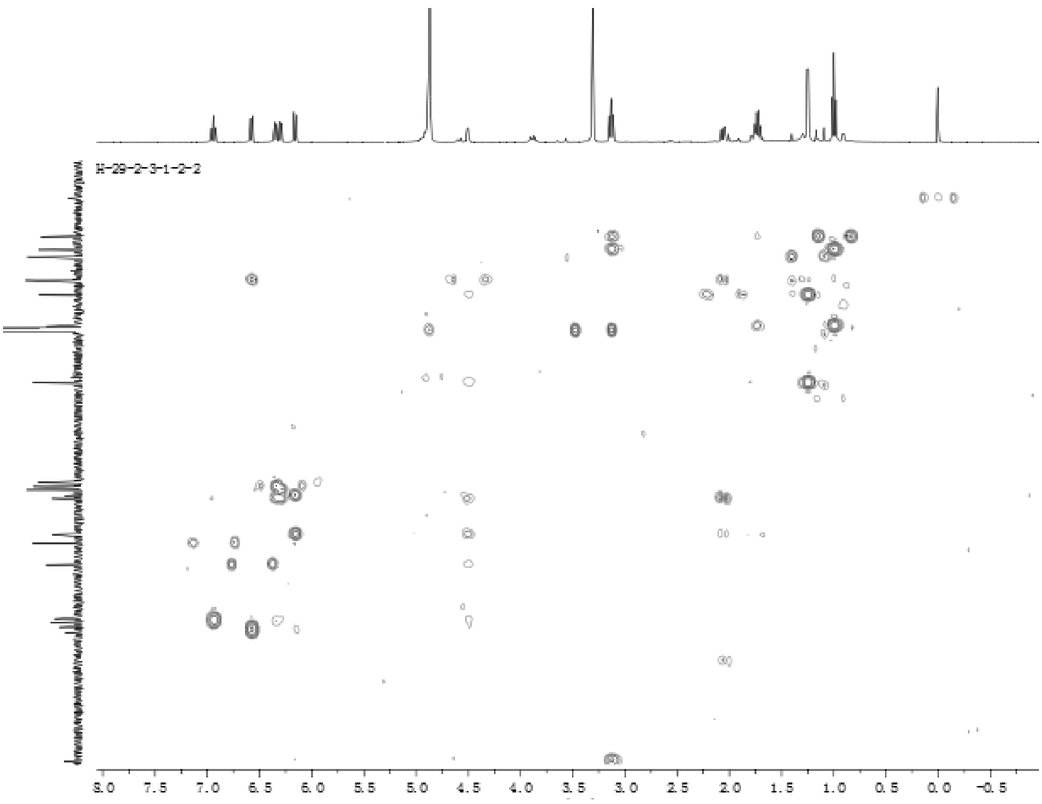

Figure S21 HMBC spectrum of **3**.

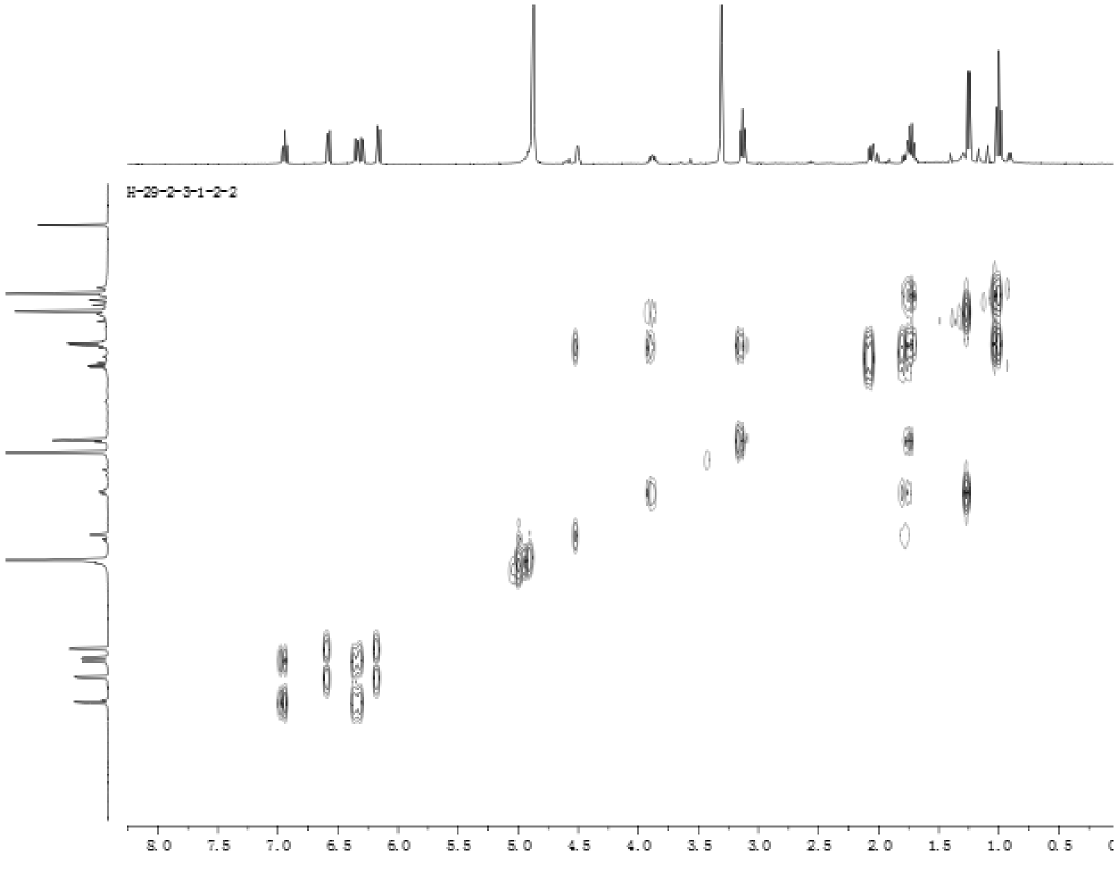

Figure S22 COSY spectrum of **3**.

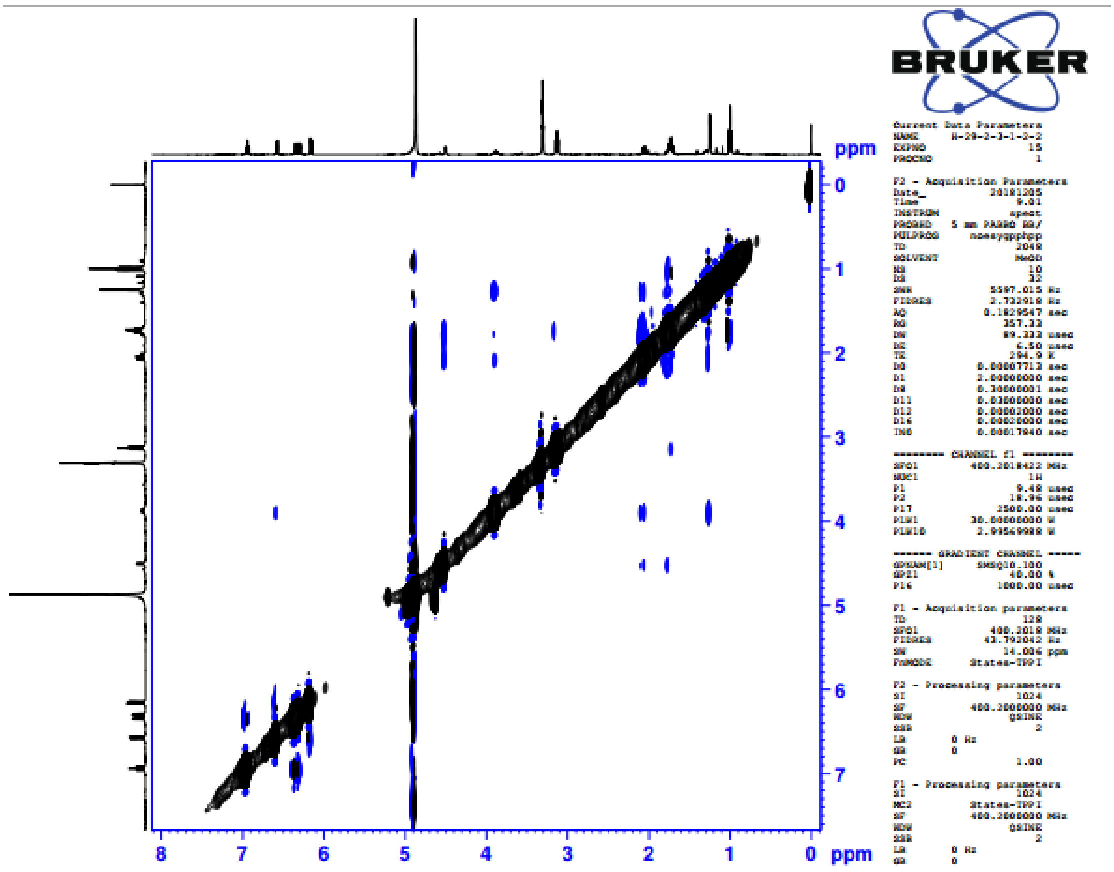

Figure S23 NOESY spectrum of **3**.

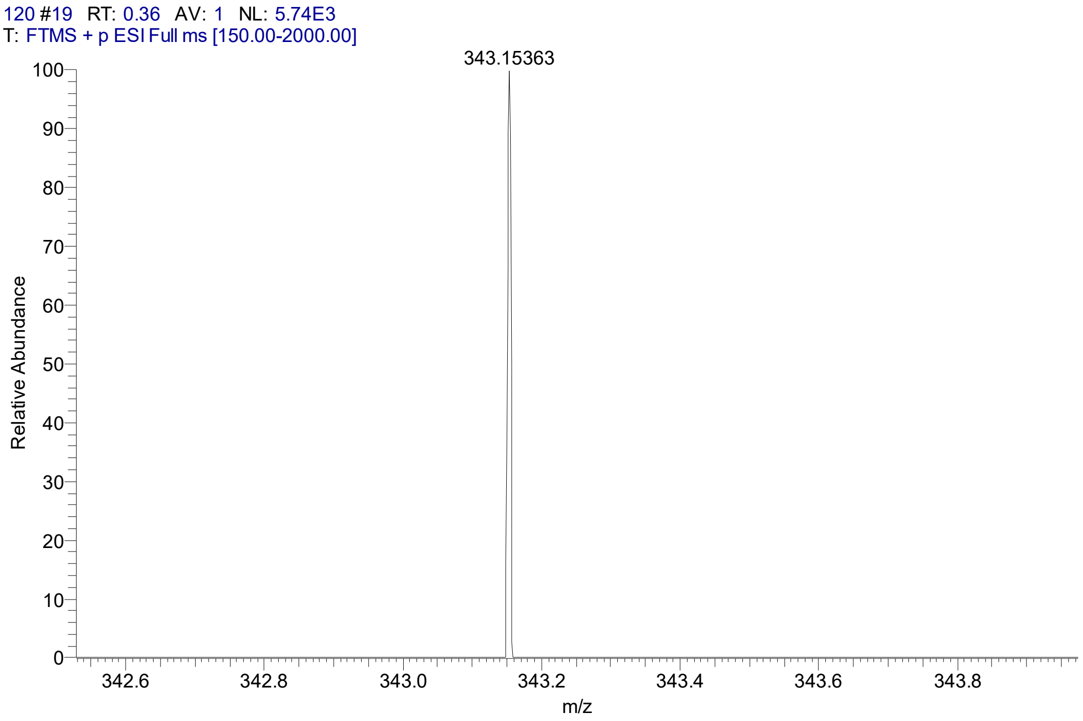

Figure S24 HRESIMS spectrum of **3**.

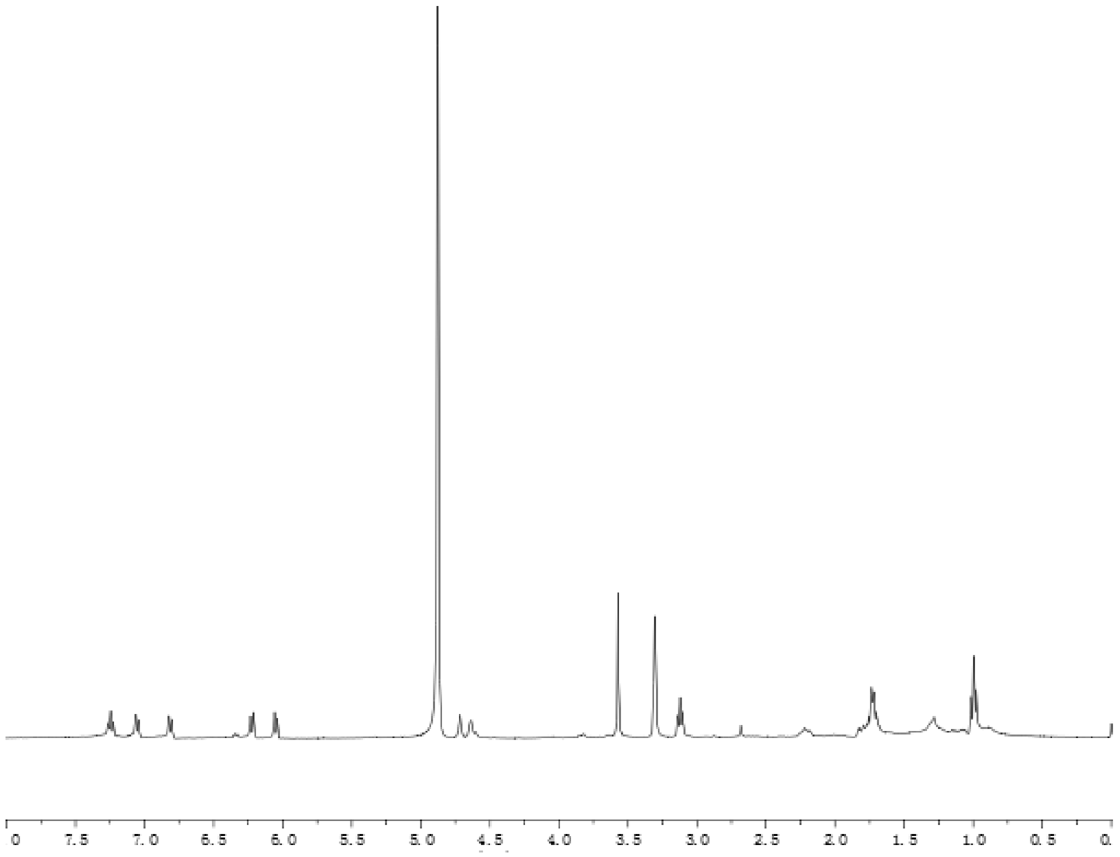

Figure S25. ^{1}H NMR (CD_3OD, 400 MHz) spectrum of **4**.

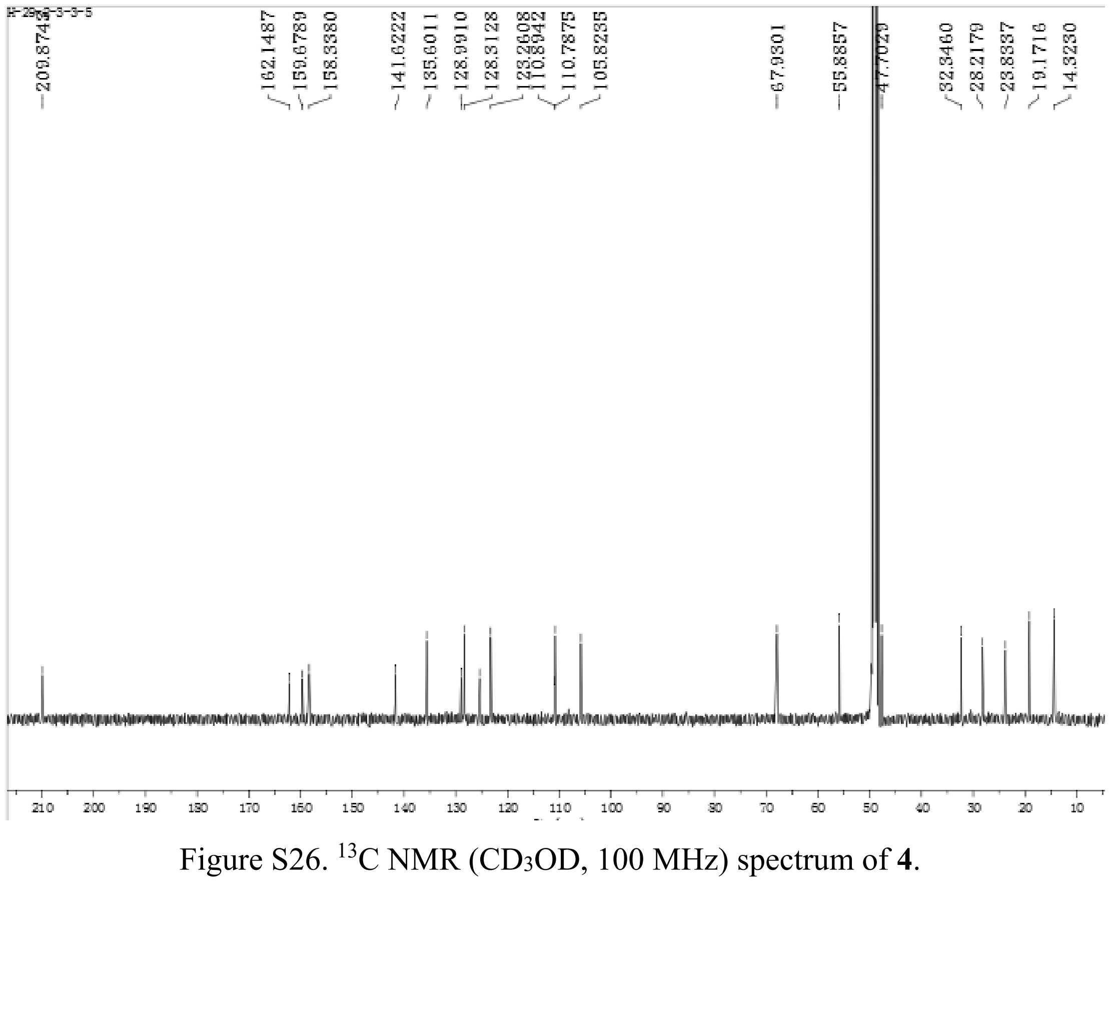

Figure S26. ^{13}C NMR (CD_3OD, 100 MHz) spectrum of **4**.

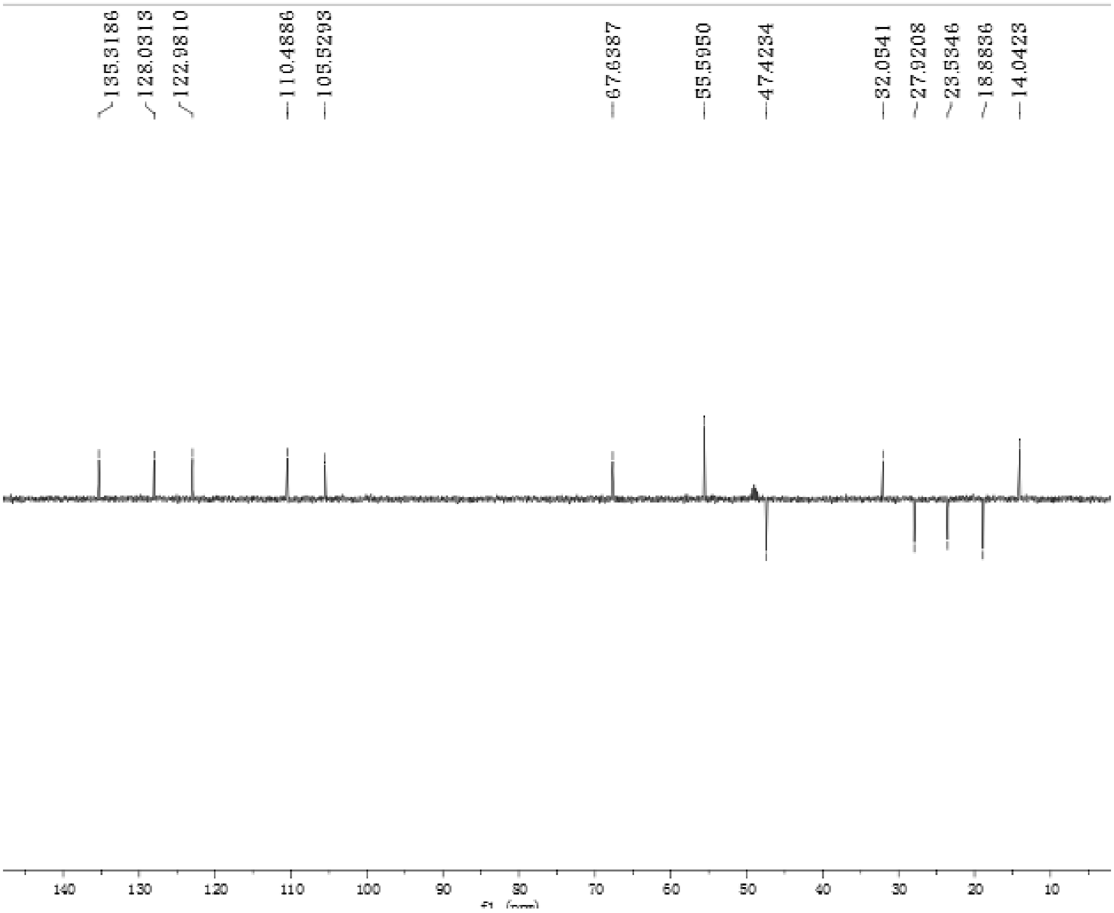

Figure S27. DEPT (CD_3OD, 100 MHz) spectrum of **4**.

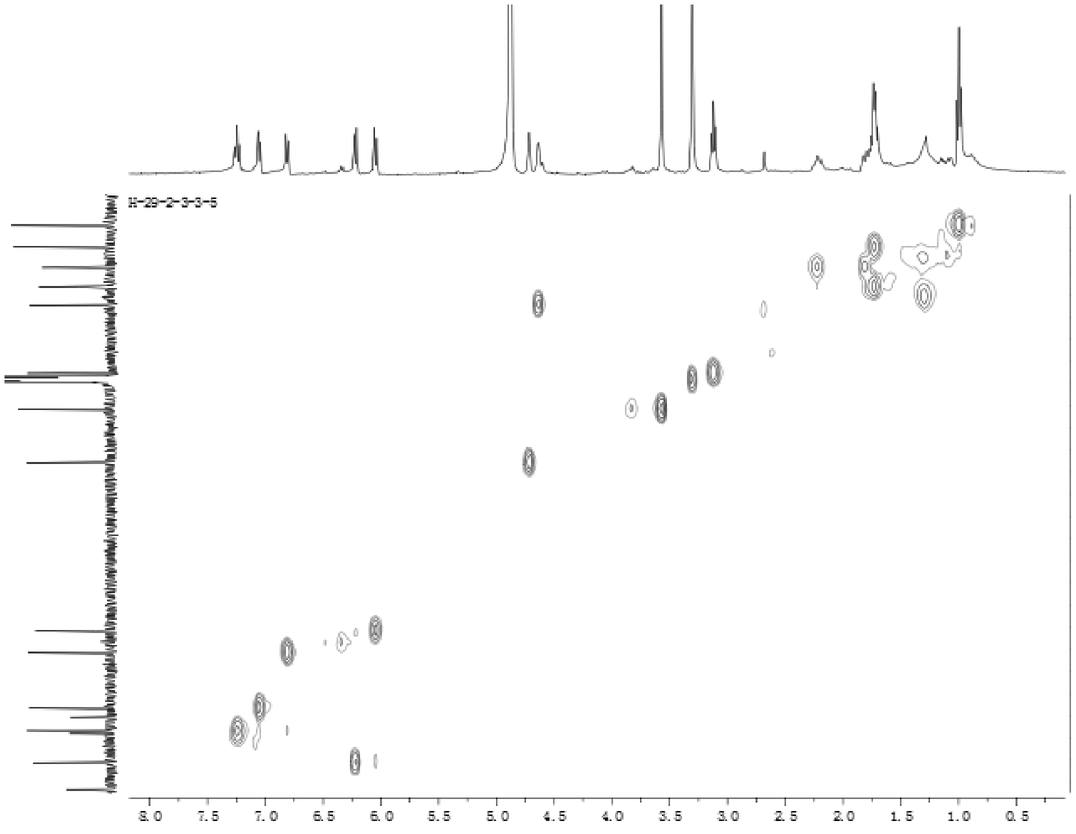

Figure S28. HMQC spectrum of **4**.

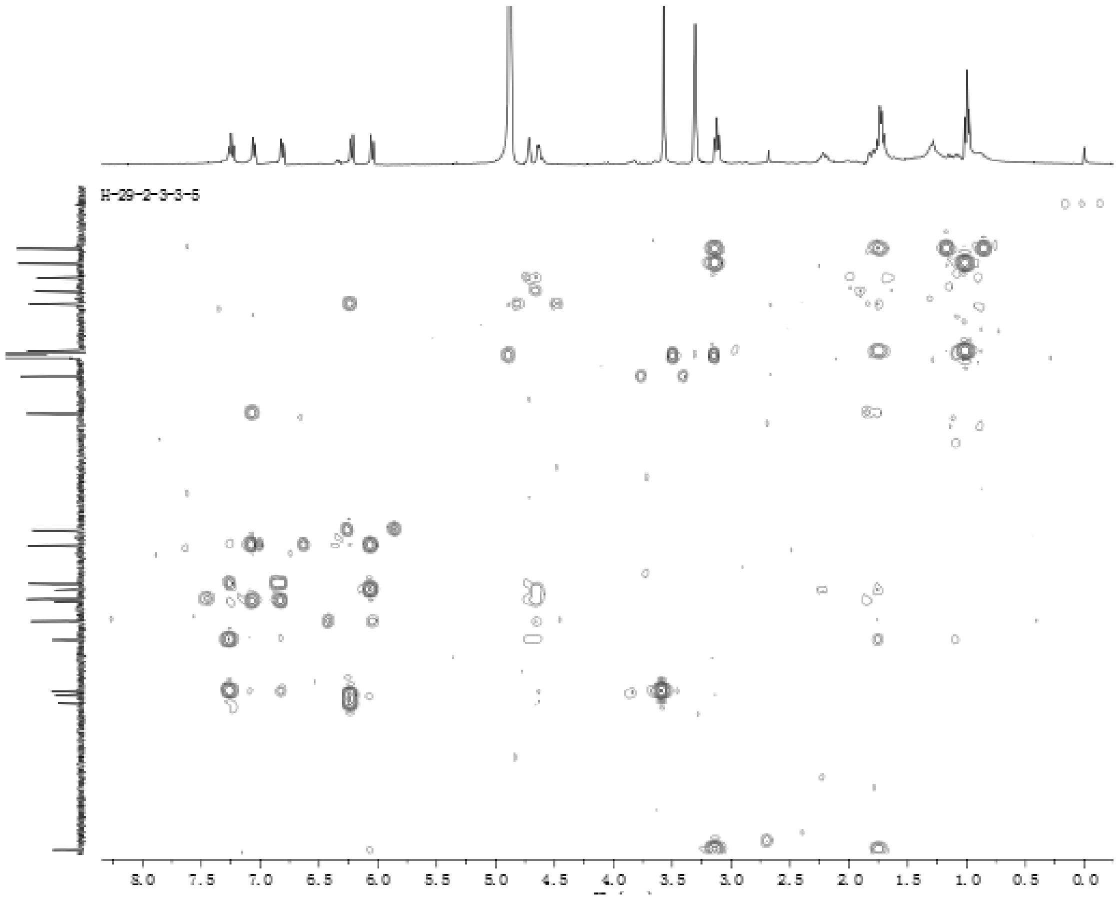

Figure S29. HMBC spectrum of **4**.

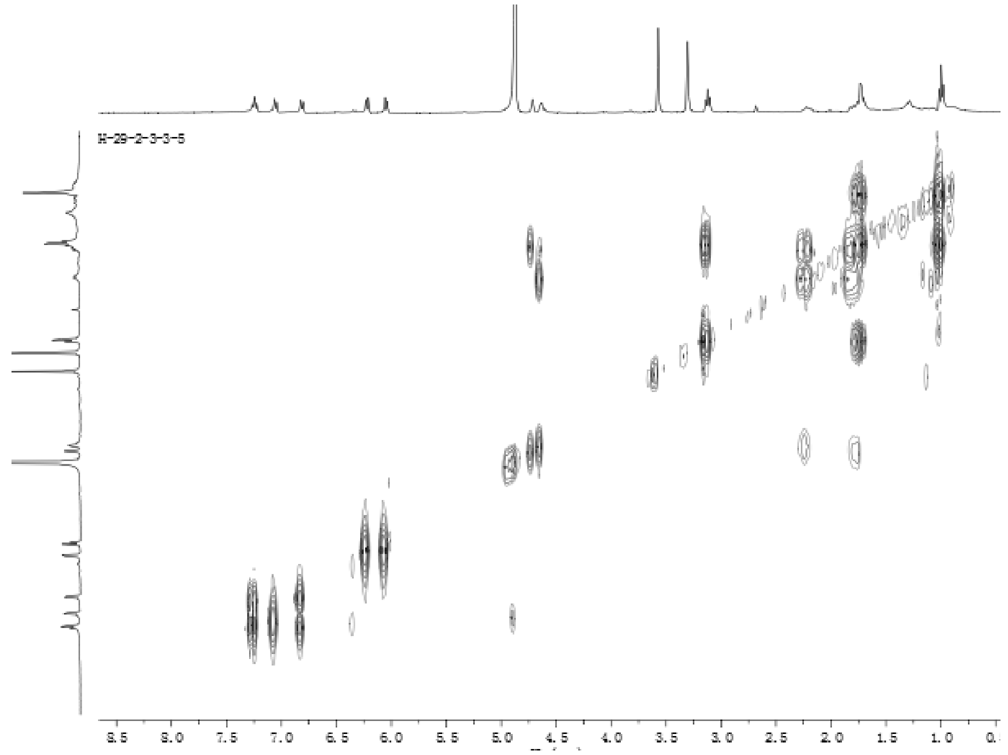

Figure S30. COSY spectrum of **4**.

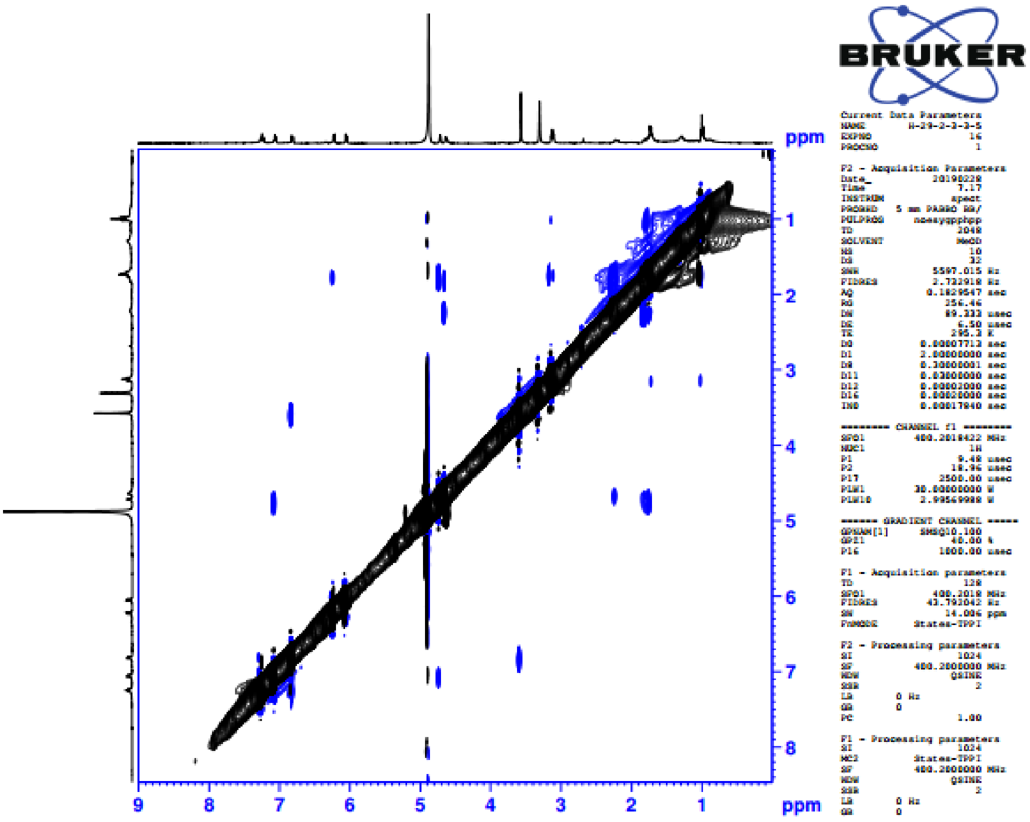

Figure S31. NOESY spectrum of **4**.

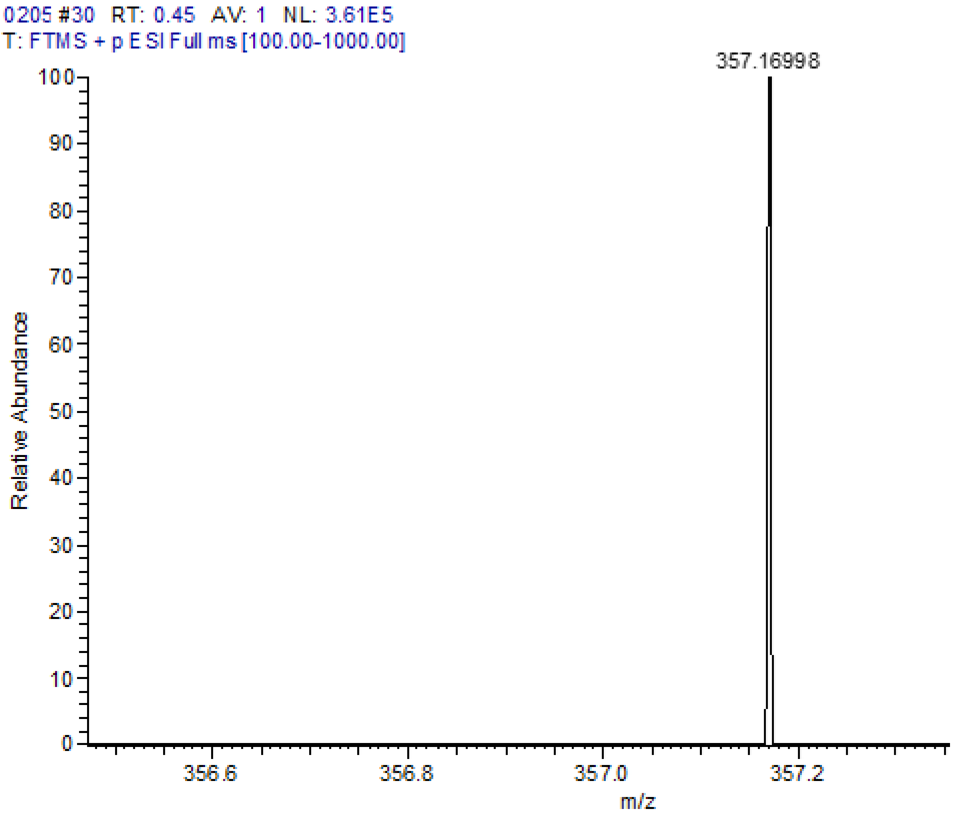

Figure S32. HRESIMS spectrum of **4**.

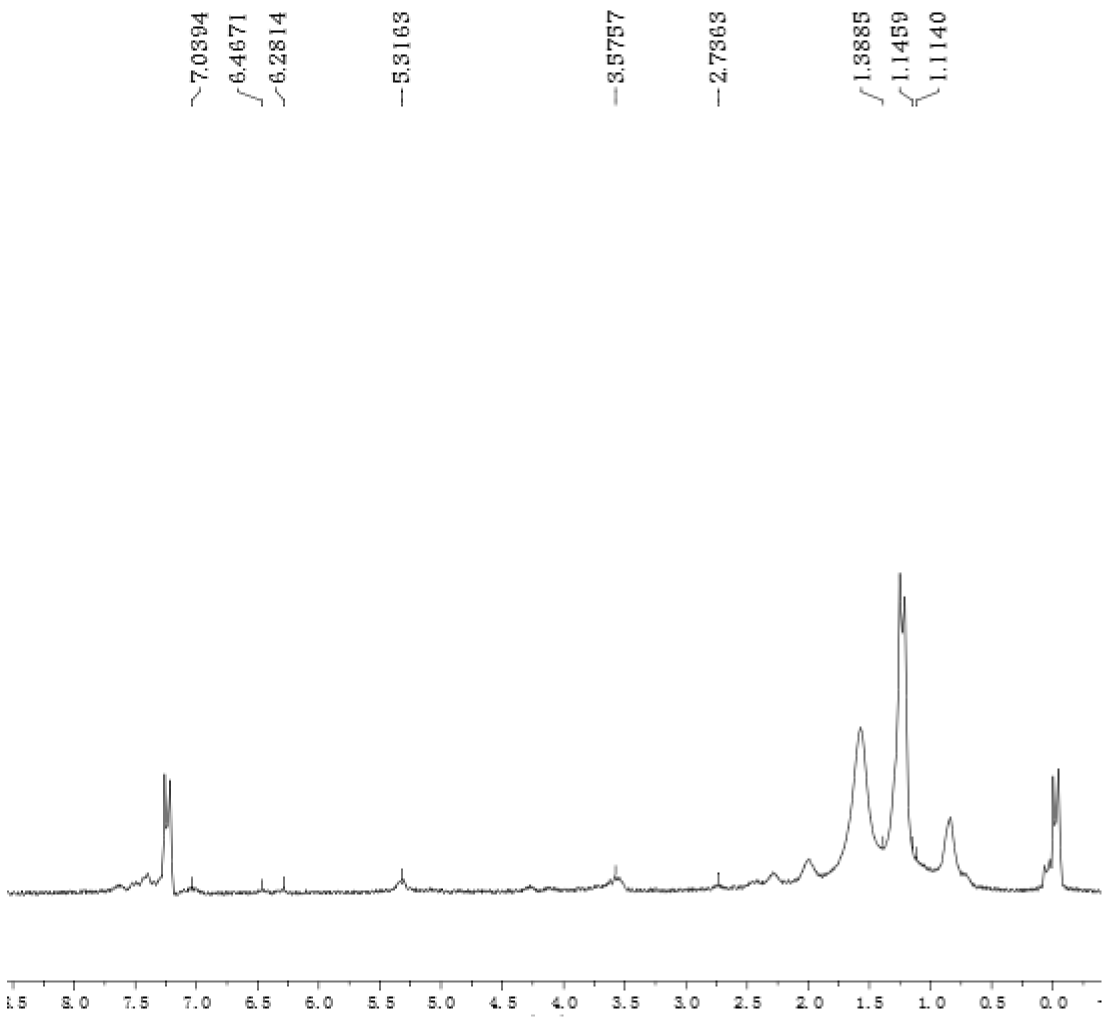

Figure S33. ^{1}H NMR ($CDCl_3$, 400 MHz) of *S*-MTPA ester of **2a**

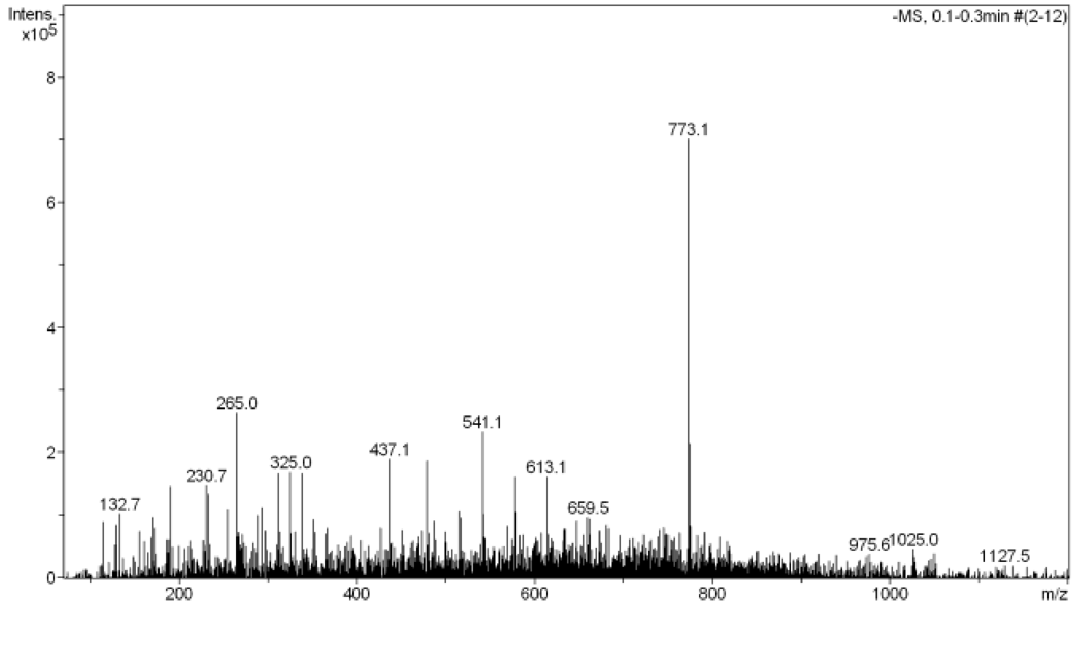

Figure S34. ESI-MS spectrum of **2a**

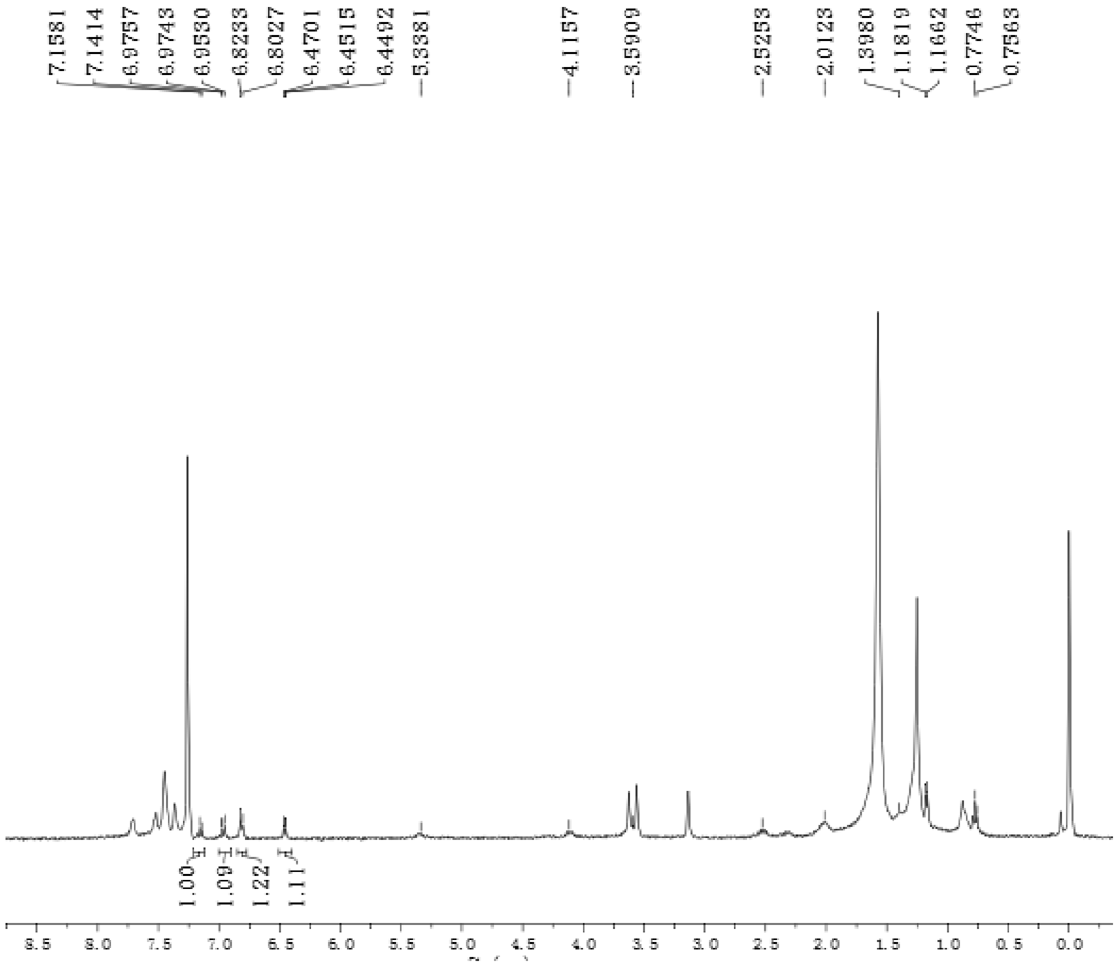

Figure S35. ^{1}H NMR ($CDCl_3$, 400 MHz) of *R*-MTPA ester of **2b**

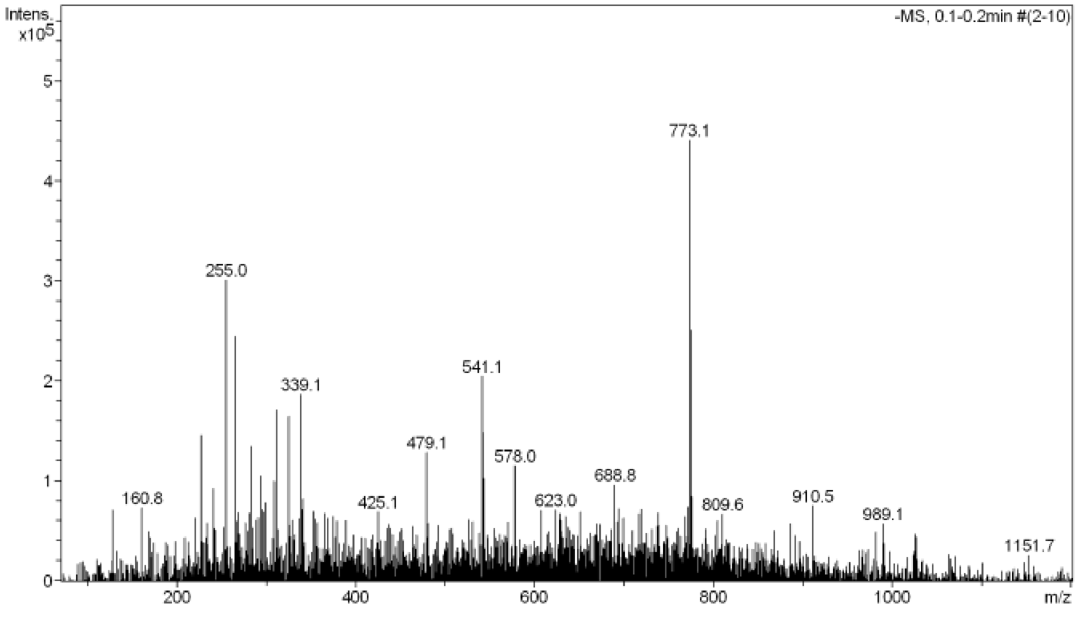

Figure S36. ESI-MS spectrum of **2b**

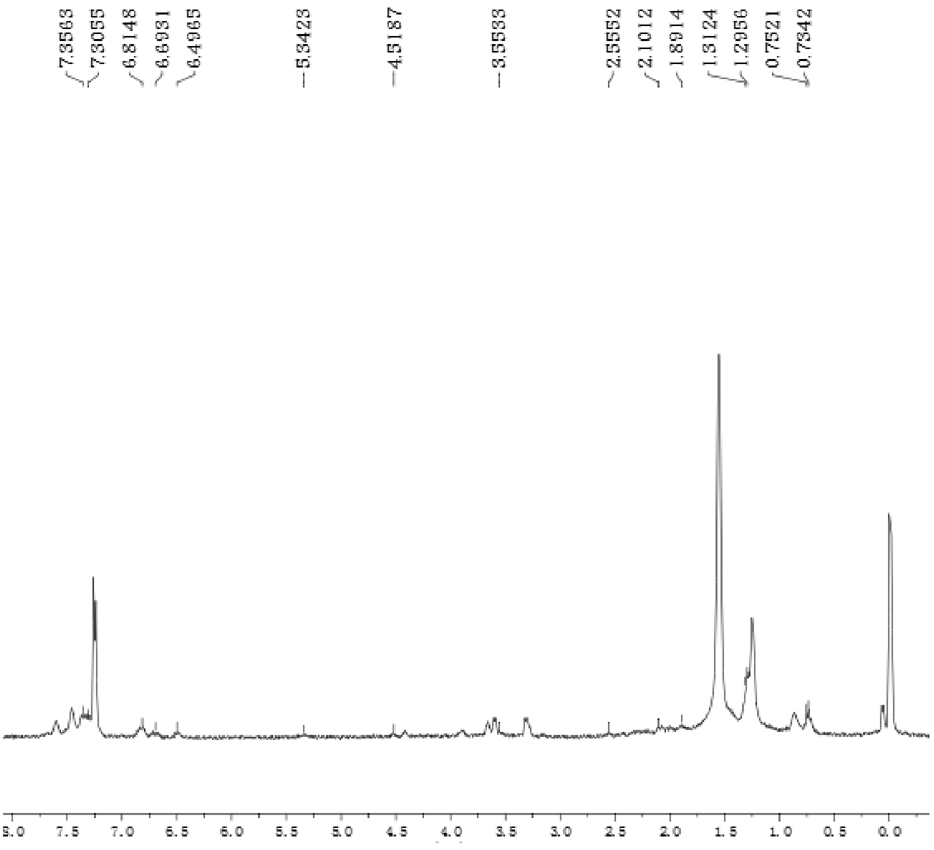

Figure S37. ^{1}H NMR ($CDCl_3$, 400 MHz) of *S*-MTPA ester of **3a**

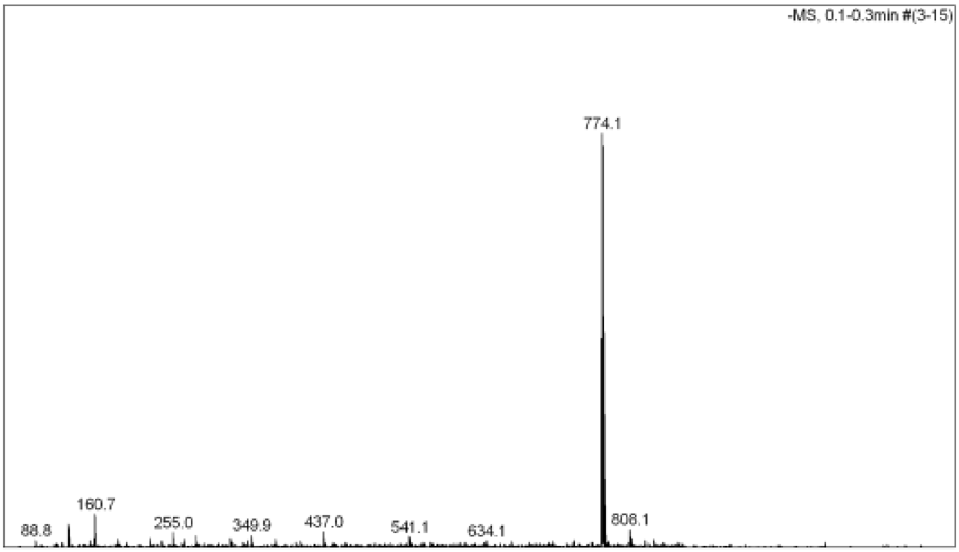

m/z	z	I	FWHM
128.7		152169	0.2
160.7		217463	0.2
255.0		96911	0.3
437.0		101587	0.2
773.2		1363135	0.3
774.1		2709062	0.3
775.1		2626367	0.3
776.1		1410613	0.3
777.1		446813	0.3
808.1		117650	0.2

Figure S38. ESIMS spectrum of **3a**

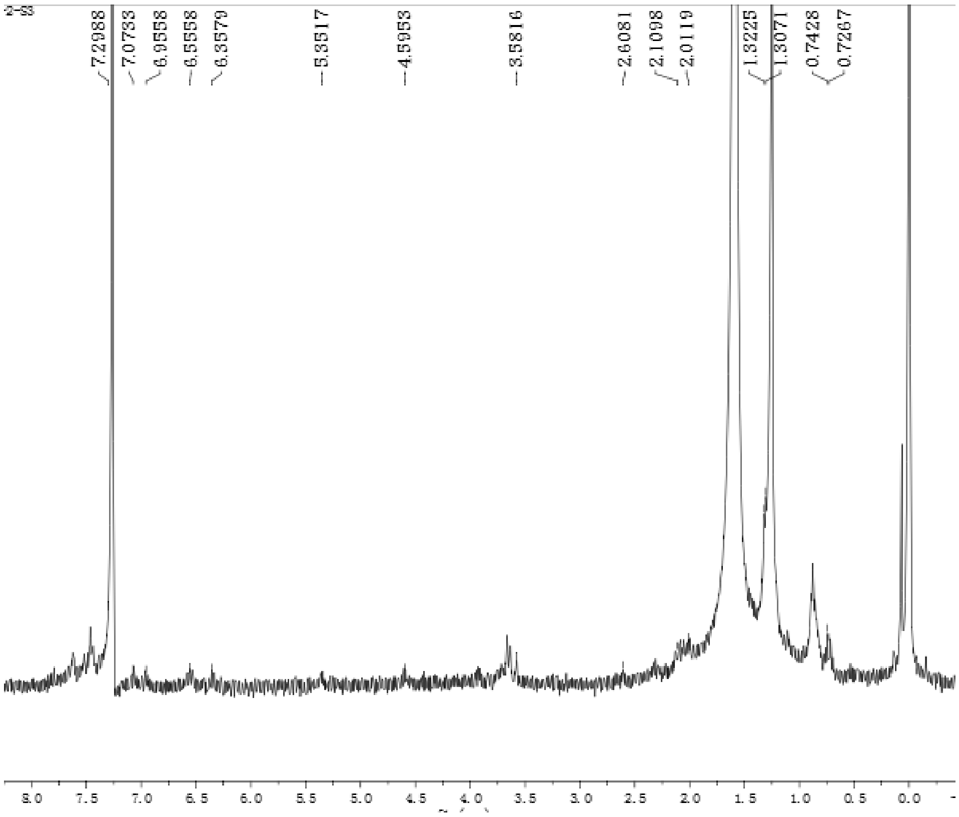

Figure S39. ^{1}H NMR ($CDCl_3$, 400 MHz) of *R*-MTPA ester of **3b**

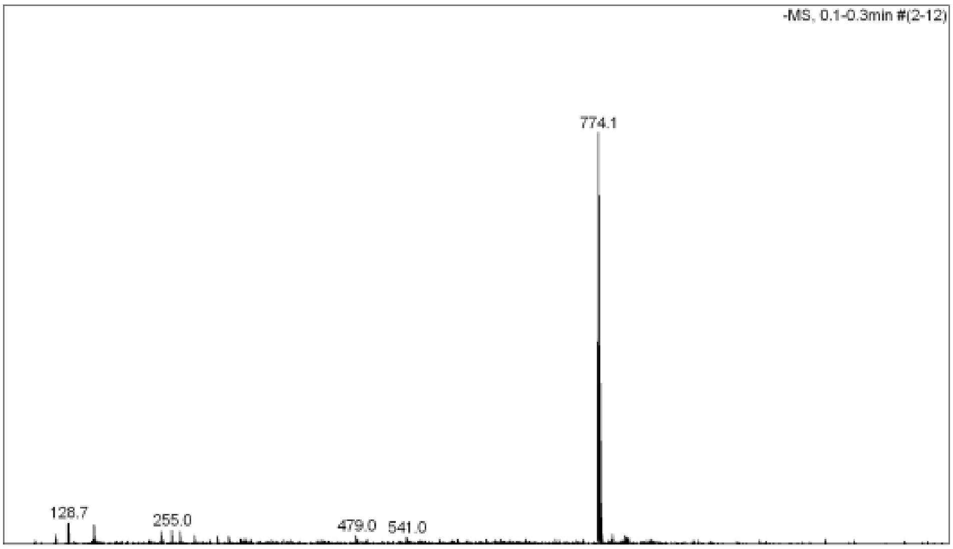

m/z	z	I	FWHM
128.7		142748	0.2
160.7		129020	0.2
241.9		82891	0.3
255.0		93321	0.3
264.9		81494	0.2
773.1		1335563	0.3
774.1		2716571	0.3
775.1		2302053	0.3
776.1		1062379	0.3
777.1		269089	0.3

Figure S40. ESIMS spectrum of **3b**
